# Asymmetric transmission in nanophotonics

**DOI:** 10.1515/nanoph-2022-0820

**Published:** 2023-04-10

**Authors:** Abbas Sheikh Ansari, Ashwin K. Iyer, Behrad Gholipour

**Affiliations:** Department of Electrical and Computer Engineering, University of Alberta, Edmonton, Canada

**Keywords:** asymmetric transmission, magneto-optic, metagrating, metamaterials and metasurfaces, nonreciprocal transmission, reciprocal transmission

## Abstract

In a reciprocal medium, transmission of electromagnetic (EM) waves is symmetric along opposite directions which restrict design and implementation of various systems in optics and photonics. Asymmetric transmission (AT) is essential for designing isolators and circulators in optics and photonics, and it benefits other applications such as photovoltaic systems, lasers, cloaking, and EM shielding. While bulky nonreciprocal devices based on magnetic field biases have been well known, creating AT in subwavelength structures is more challenging, and structures with a subwavelength thickness that show AT have drawn a lot of attention over the last decade. Various approaches have been reported to create metasurfaces featuring nonreciprocal transmission, such as plasmonic and dielectric metasurfaces that enhance Faraday rotation, nonlinear metasurfaces with intensity-dependent refractive indices, and implementing spatiotemporal modulation in a metasurface. On the other hand, AT has also been reported in reciprocal structures by creating multiple paths for the transmission of EM waves by changing the polarization of light or redirecting light to higher-order diffraction orders. Here, we present a review of various approaches implemented for realizing AT in subwavelength structures in both reciprocal and nonreciprocal systems. We also discuss the main design principles and limitations of AT achieved in various approaches.

## 1 Introduction

Developments in nanofabrication technologies in the last two decades have revolutionized the field of optics and photonics by enabling the realization of nanostructures. Arrangements of subwavelength nanostructures with arbitrary geometries, also known as metamaterials and metasurfaces [1–3], provide new possibilities for controlling light not feasible in conventional optics. Manipulating light–matter interaction on the subwavelength level can be used to control spatial and temporal distribution of electromagnetic (EM) waves and can lead to designing compact devices that change the direction of propagation, polarization, phase, frequency and intensity of EM waves. Metasurfaces can be used in free-space optical systems, such as Lidar and Lifi systems, 3D holograms, and compact imaging systems [4–6]. Furthermore, metasurfaces can be integrated with on-chip photonic waveguides and optical fibers, which benefit optical communication systems and on-chip photonic computing [7–9]. Engineered subwavelength structures can also create strong resonances that significantly increase the intensity of light and enhance the light–matter interactions, which benefit various applications related to sensing, nonlinear optics, laser applications, and quantum optics.

One important class of structures developed for controlling EM waves are those providing asymmetric transmission (AT). A device that supports AT has different responses for excitation from different directions such that it allows transmission of the EM waves to desired angles for excitation from one side, but it has a negligible transmission to the same angles for excitation from the other side. AT is important for creating isolation among different components in optical and microwave communication systems, it is useful for decoupling different paths of light in quantum optics applications, and it benefits applications where EM shielding and cloaking are required [10–13]. Besides, it can enhance the efficiency in solar cell systems by trapping light and enhancing the absorption for solar cells.

AT has long been studied in photonic circuits and microwave waveguides for designing isolators and circulators. Since the advent of metasurfaces, AT of free-space waves has attracted a lot of attention, and various approaches are suggested for realizing AT in thin structures. Generally, AT of free-space waves from a planar optically thin structure is more challenging due to the limited interaction range of EM waves with matter.

AT has been used for both reciprocal and nonreciprocal structures in the literature. However, reciprocal AT and nonreciprocal transmission are quite different with distinct advantages and limitations. Here, we review AT in reciprocal and nonreciprocal structures to clarify the differences between these two and identify the applications for each scenario.

Reciprocity stems from the time-reversal symmetry of Maxwell equations meaning that if one considers the propagation and scattering of EM waves as a movie, then playing that movie backward also is a valid solution for wave equations [12, 14, 15]. In any EM scattering problem, reciprocity implies that if an arbitrary incident field *E*_i_ creates a scattered field *E*_s_, then considering *E*_s_ as an incident wave must result in *E*_i_ as the scattered field.

Nonreciprocal transmission requires breaking the time-reversal symmetry. Traditionally, materials showing considerable magneto-optic effect such as yttrium iron garnet (or cerium- or bismuth-substituted yttrium iron garnet) have been used to achieve AT based on Faraday rotation [10, 11, 16], [17], [18], [19], [20], [21]. Although isolators and circulators based on magnetic materials are available as discrete optical components, integrating such components with photonic circuits and designing compact isolators for free space waves has been more challenging [10]. Faraday rotation in EM fields depends on the strength of the magnetic field bias and the distance along which the wave interacts with the magnetic field. Thus, for free space planar structures where the interaction between matter and EM fields occurs over short distances, one cannot achieve a considerable amount of Faraday rotation. To overcome this limitation, metasurfaces supporting plasmonic resonances [22–31] and Mie resonance [32–34] have been proposed to enhance the field-matter interaction and increase the Faraday rotation in planar structures.

The existence of magnetic field bias and materials with magnetic properties is not desirable for several applications where AT is required. One approach for achieving nonreciprocal transmission without the presence of a magnetic field is to use a nonlinear system. In an asymmetric geometry, the intensity of the EM field at a fixed point is not the same for excitation from different sides. On the other hand, in a nonlinear medium, the effective permittivity depends on the EM field intensity in different regions. Thus, in an asymmetric structure involving a nonlinear material, the effective permittivity distribution varies for excitation from different sides. Materials showing a considerable amount of optical nonlinearity in the presence of a strong electric field have been used to create magnet-free nonreciprocal transmission [35–43] for waveguides [35–39], and free space waves [41–43]. Similar principles in volatile phase change materials [44, 45], optomechanical nonlinear metamaterials [46, 47], and optomechanical coupled resonators [48, 49] can provide AT. The major limitation for these structures is that they can create AT only for high input powers, and they cannot operate when the structure is excited from both sides simultaneously [50].

Another approach for breaking time-reversal symmetry and creating nonreciprocal transmission is to use spatiotemporal modulation [51–69]. As the simplest incaranation of such a system, one can consider a dielectric slab moving up (or down) with a constant speed ${\overset{⇀}{v}}_{0}$ with respect to an inertial frame of reference. In this case, as we know from the Doppler effect, the propagation constant of the wave propagating in the same direction as ${\overset{⇀}{v}}_{0}$ is different from the one propagating in the opposite direction, which can result in different transmissions. A more realistic scenario is using spatiotemporal modulation of permittivity. Various methods have been reported for realizing spatiotemporal modulation in waveguides and photonic circuits [51, 53], [54], [55], [56], [57]. In the case of metasurfaces, spatiotemporal modulation has been achieved with the combination of gradient metasurfaces and time modulation of individual unit cells. For these structures, leaky waves propagating along the surface are essential to provide the desirable spatiotemporal modulation. The major limitation in using spatiotemporal structures for achieving AT is that usually, the required modulation frequency for optical frequencies is larger than what can be achieved with well-known electro-optic modulators.

Aside from nonreciprocal transmission, AT has also been reported in reciprocal structures. Reciprocal AT relies on designing structures that can create multiple paths for an incident wave such that excitation from different sides couples the input energy to different channels. The major limitation in reciprocal AT is that one can create asymmetry in the transmission for a specific type of excitation. For example, one can generate AT for a specific polarization or a specific direction of the incident wave.

Two polarization states of light can create different paths for transmission and generate AT. Chiral and bianisotropic metamaterials and metasurfaces have been extensively studied for achieving polarization-based AT. In chiral media, the propagation of the right-handed circularly polarized wave (RCP) and left-handed circularly polarized (LCP) waves are different, and one can design such structures in a way that RCP (or LCP) has a high transmission, and the other polarization has negligible transmission. Chiral metamaterials composed of helixes [70], and multilayer chiral metasurfaces have been demonstrated for achieving polarization-based AT [71–75]. Furthermore, single layer metasurfaces comprising unit cells with multiple resonators with different sizes and orientations has been proposed for achieving AT metasurfaces [76, 77].

Another approach for creating multiple channels for the incident wave is based on exploiting diffraction orders. AT based on coupling the incident energy to different diffraction orders, first has been studied in photonic crystal slabs with different corrugations on opposing sides [78–80]. The periodicity of the corrugations on two sides of the photonic crystal slab is different such that an incident wave with a fixed angle can couple to one of the propagating modes inside the slab from one side only. Indeed, any periodic structure that can couple the incident energy to higher diffraction orders and lacks mirror symmetry along the propagation of the incident wave can create AT. Several structures such as double layer gratings with different periodicities [81–85], asymmetric 1-D [86, 87], and 2-D metasurfaces [88–90], composition of gradient metasurfaces and gratings [91, 92], hyperbolic metamaterials [93] and photonic crystals waveguides [94–101] have been proposed for creating AT based on diffraction orders.

This paper is divided into three parts. In Section 2, we discuss nonreciprocal transmission. In this part, we start with a quick review of the nonreciprocity principle and then discuss nonreciprocal transmission based on the magneto-optic effect. We then review power-dependent nonreciprocal transmission metasurfaces using nonlinear materials and different spatiotemporal modulations used for breaking the reciprocity. In Section 3, we review various methods that have been used for achieving reciprocal AT. First, we review AT metasurfaces working based on polarization conversion principles, and next, we discuss AT based on diffraction orders in periodic structures. The last part discusses the potential application for different scenarios and outlook of reciprocal and nonreciprocal AT.

## 2 Nonreciprocal transmission

### 2.1 Nonreciprocity principles

Reciprocity is a fundamental theorem in electromagnetics, and it leads to certain symmetries in scattering of light. Reciprocity causes symmetric transmission from opposite directions in most waveguide and free space structures which is not desirable for various applications, and extensive research has been done toward breaking the reciprocity and achieving AT. Reciprocity can be best understood in terms of the time-reversal symmetry in EM waves and breaking this symmetry is the key to achieving nonreciprocal transmission.

Most physical phenomena are time-reversal symmetric, meaning that they do not depend on the direction of time. For any physical quantity that evolves over time from an initial state at *t* = *t*_0_, and reaches another state at time *t* = *t*_2_, applying time-reversal operation means reversing the direction of the time to see how that quantity changes in the reverse direction as time changes from *t* = *t*_2_ to *t* = *t*_0_. Mathematically, when time-reversal operator, F , acts on a physical quantity at time *t*_1_, it results in the value of that quantity at −*t*_1_. Accepting the fact that electric charge is symmetric under time reversal operation, one can deduce the symmetry or asymmetry of other electromagnetic quantities such as current density, electric field and magnetic field under the time-reversal operator. One can show that electric field has an even symmetry under time reversal operation, $F\left\{E\left(x,t\right)\right\}=E\left(x,-t\right)$ , and magnetic field has odd symmetry, $F\left\{H\left(x,t\right)\right\}=-H\left(x,-t\right)$ . Phase velocity and propagation vector as well as the Poynting vector have odd symmetry under time reversal operators. Therefore, time-reversal operator reverses the direction of propagation in EM waves, i.e., time-reversal symmetry in EM waves results in reciprocity of EM waves. In systems without loss and gain, reciprocity and time reversal symmetry are equivalent. In systems with loss and gain, definition of time-reversal needs to become more general to become equivalent with reciprocity. There are several representations and formulations for the reciprocity principle. Onsager showed the reciprocity principle based on the microscopic interactions in terms of polarization tensors that relate electric and magnetic dipole moments to EM fields in matter [102]. On the other hand, Lorentz reciprocity theorem expresses the reciprocity for macroscopic quantities by considering two sources and the electric field they produce in space. If one considers two current sources *J*_A_, and *J*_B_, in a fixed problem, and these sources create fields *E*_A_, and *E*_B_ in space, then according to the Lorentz reciprocity theorem we will have: ∫*J*_A_ ⋅ *E*_B_d*v* = ∫*J*_B_ ⋅ *E*_A_d*v*.

Where the integral is over the whole space. One can show that this relation can be satisfied in a linear time-invariant medium provided that permittivity and permeability tensors are symmetric, meaning that ${\bar{\bar{\epsilon }}}^{T}=\bar{\bar{\epsilon }}$ and ${\bar{\bar{\mu }}}^{T}=\bar{\bar{\mu }}$ where superscript *T* represents the transpose. This is an important conclusion that we use for our discussion about bianisotropic metamaterials and metasurfaces. Such structures can be modeled with permittivity and permeability matrices which have nonzero off-diagonal elements but are symmetric under transpose operation. The presence of off-diagonal elements could result in polarization control and creating polarization-based reciprocal AT.

Another representation of the reciprocity principle is based on symmetries of the scattering matrix. EM fields in any structure can be written based on a superposition of all the modes in the problem. When we have a finite number of modes that interact with each other in the system, a scattering matrix can represent all the information in the problem. For such systems, one can show that reciprocity results in a symmetric scattering matrix. This representation is useful for discussing reciprocal AT based on multiple diffraction orders in periodic structures as will be discussed in Section 2.2.

Although all the representations refer to the same principle, each of them analyses the problem from a different perspective, and understanding various approaches helps us to find new methods for achieving AT.

The time reversal symmetry in EM fields arises from the fact that replacing *t* with −*t* results in changing the direction of the velocity and momentum. Using a magnetic field bias or using an external force that creates a time-varying medium can therefore break the symmetry in the momentum in microscopic interactions and break the reciprocity.

Here we classify into three categories the different approaches for breaking the time reversal symmetry: (A) magnetic field bias, (B): Intensity dependent refractive index, and (C) spatio temporal modulation.

**Table 1:** Transformation of electrodynamic quantities under time reversal operator.

Physical quantity	Time reversal F{}
Charge density *ρ*(*t*)	$F\left\{\rho \left(t\right)\right\}=\rho \left(-t\right)$
Current density *j*(*t*)	$F\left\{j\left(t\right)\right\}=-j\left(-t\right)$
Electric field $E\left(t\right)$	$F\left\{E\left(t\right)\right\}=E\left(-t\right)$
Magnetic field $B\left(t\right)$	$F\left\{B\left(t\right)\right\}=-B\left(-t\right)$
Electric polarization density, *P*(*t*)	$F\left\{P\left(t\right)\right\}=P\left(-t\right)$
Poynting vector *S*(*t*)	$F\left\{S\left(t\right)\right\}=-S\left(-t\right)$

### 2.2 Nonreciprocity based on magnetic field bias

To investigate the origin of the nonreciprocity in the presence of a magnetic field bias, first we consider the interaction of a single charged particle with EM fields in the presence of a magnetic field bias. In this system, the total force acting on the particle is ***F*** = *q* ***v*** × ***B***_0_ + *q* ***E***, where ***v*** is the particle velocity, and *q* is the electric charge, (all bold symbols represent a vector). After applying the time reversal symmetry operation on this equation (Table 1), the direction of the velocity changes while the magnetic field bias is fixed, so the first term on the right-hand side becomes negative. Electric field and charge are symmetric under time reversal symmetry, so the second term does not change. Therefore, in the presence of a magnetic field bias the total force acting on a charged particle is not time reversal symmetric, leading to nonreciprocity. We should note that usually, the effect of the magnetic field corresponding to the EM waves is negligible with respect to the force from the electric field, but even considering the force from the magnetic field in the EM wave in the absence of a magnetic field bias does not break the reciprocity since a magnetic field has an odd symmetry under time reversal operator which cancels out with odd symmetry of the velocity vector.

This argument can be generalized from interaction with a single particle to interaction with a solid material by considering a simple model for electric dipole moment in the material. We consider the electric dipole in the classical regime as a pair of particles with opposite charges where the position of the positive charge is fixed, and we approximate the effective potential of an electron with a harmonic oscillator model. For an arbitrary potential, this model corresponds to the first term in its Taylor expansion. The electron’s position can be described as:

(1) $$m\frac{\partial^{2}r}{\partial t^{2}}+m\bar{\bar{\Omega }}r-\bar{\bar{\Gamma }}\frac{\partial r}{\partial t}=q\frac{\partial r}{\partial t}\times B_{0}+qE$$

In general, $\bar{\bar{\Omega }}$ is a matrix with Ω_ij_ = ∂*V*/∂*x*_*i*_∂*x*_*j*_. However, we can rotate the coordinate system such that $\bar{\bar{\Omega }}$ becomes diagonal. $\bar{\bar{\Gamma }}$ is also a diagonal matrix for modeling the loss because of collisions in the system. Rewriting this equation in the frequency domain, we have:

(2) $$\left(-m\omega^{2}I+m\overset{¯}{\overset{¯}{\Omega }}-\text{i}\omega \overset{¯}{\overset{¯}{\Gamma }}\right)r=\text{i}q\omega r\times B_{0}+qE$$

Solving this matrix equation results in the position of the electron, and the electric dipole moment **p** = *q* **r**. From this, one can calculate polarizability coefficients from $p=\bar{\bar{\alpha }}E$ and calculate electric permittivity from there. For diagonal $\bar{\bar{\Omega }}$ and $\bar{\bar{\Gamma }}$ matrices in Eq. (2), **r** × **B**_0_ term results in the appearance of non-equal off-diagonal components for polarizability coefficients such that it leads to a non-symmetric permittivity tensor $\left(\bar{\bar{\varepsilon }}\neq {\bar{\bar{\varepsilon }}}^{T}\right)$ and this breaks the Lorentz reciprocity condition. If we consider a symmetric potential such that $\bar{\bar{\Omega }}=\left(\begin{array}{cc} \omega_{0}^{2} & 0\\ 0 & \omega_{0}^{2} \end{array}\right),\bar{\bar{\Gamma }}=\left(\begin{array}{cc} \gamma & 0\\ 0 & \gamma \end{array}\right)$ , polarizability components can be calculated from (2) and one can show that:

(3) $$\alpha_{xy}=-\alpha_{yx}=\text{i}\frac{q^{2}}{\varepsilon_{0}}\left(\frac{\left(\omega B_{0}q\right)}{{\left(-m\omega^{2}+m\omega_{0}^{2}-\text{i}\omega \gamma \right)}^{2}-{\left(\omega B_{0}q\right)}^{2}}\right)$$

**Figure 1:**
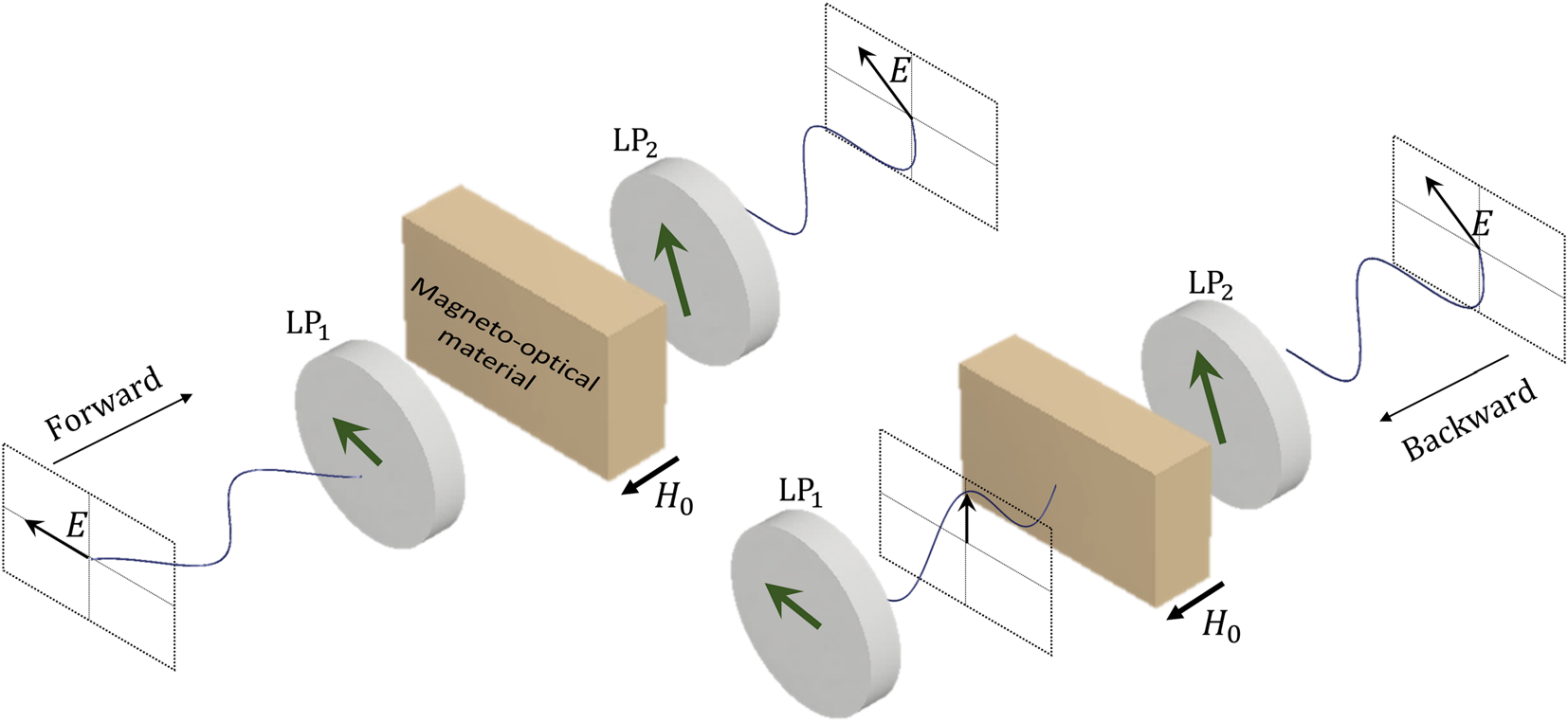
Combination of Faraday rotation and polarizer for nonreciprocal transmission.

Thus, one can see that for this example, the polarizability tensor has off-diagonal elements with opposite signs, violating the Lorentz reciprocity theorem. When the polarizability tensor and its corresponding permittivity tensor have off-diagonal elements with opposite signs, different polarizations of light propagate with different speeds. For a simple model we described here, one can show that left-handed circularly polarized light and right-handed circularly polarized light are eigen polarizations in the system with different effective indices. When a linearly polarized light transmits through such a material, the polarization vector rotates, which is known as Faraday rotation. Using two linear polarizers on two sides of a material having magneto-optic effect can result in AT, as shown in Figure 1. One should note that a reciprocal chiral structure can also result in polarization rotation. But in a chiral structure, polarization rotation for waves propagating in opposite directions cancels each other out.

The magnitude of Faraday rotation is the key parameter for achieving nonreciprocal transmission in such structures. Faraday rotation depends on intrinsic properties of materials as well as the magnitude of magnetic field, and the propagation length within the material. From (3), one can show that as frequency increases, the magneto optic effect gets weaker, and for optical frequencies most materials show negligible magneto-optic effect. One can also observe that the magneto-optic effect is stronger when frequency is close to $\omega_{\text{c}}=\frac{qB_{0}}{m}$ , known as cyclotron frequency. Here, *m* is the effective mass of the electron, which considers its interaction with ions, and it is a large number such that cyclotron frequency is considerably lower than optical frequencies for feasible magnetic fields. We should also note that magnetic field flux density will be enhanced for materials with magnetic properties, resulting in higher Faraday rotation angles for a fixed frequency and bias field.

Experimentally, one can measure the Faraday rotation of any material in the presence of a magnetic field bias. The figure of merit used to describe how much a material provides Faraday rotation is called Verdet constant (*V*_c_) such that the Faraday rotation when EM wave propagate through a material for a distance *L*, in the presence of a magnetic field flux *B*_0_ is given by *θ*_F_ = *V*_c_ *B*_0_ *L*.

Generally, magnetic garnets are transparent magnetic materials that have been used for creating Faraday rotation at optical frequencies [5–11]. The most popular example of such materials is Y_3_Fe_5_O_12_ or YIG, and various doped versions of it such as Bi-substituted rare-earth iron garnet [103, 104]. Material with significant magneto-optic properties has also been realized by doping conventional semiconductors with transition metals. Terbium gallium garnet (Tb_3_Ga_5_O_12_) [105] was introduced based on this principle, and it was shown that it could create large Faraday rotation with relatively low loss. Ferromagnetic semiconductors such as gallium manganese arsenide could also be used to provide magneto-optic effects needed for AT. Achieving AT based on Faraday rotation requires materials with large Verdet constant and low loss such that light can travel through the material and have high transmission. Therefore, the ratio between Verdet constant and absorption constant $\alpha \left[\frac{1}{m}\right]$ is an important figure of merit which could help one to choose the best material for a desired application. Verdet constant and its ratio for common materials providing magneto-optic effect is shown in Table 2.

**Table 2:** Properties of some magneto-optic materials.

Material	Frequency/wavelenght range	Verdet constant (*V*_c_)[rad/T m]	Max $\left(\frac{V_{c}}{\alpha }\right)$ [rad/T]
Bi-doped YIG [3]	540–780 nm	5760–384	2
YbBi:YiG [3]	1000–1760 nm	31170–4990	5.2
Terbium gallium garnet (TGG) [106]	300 THz	36	257
Ce: TGG [105]	500–1500 nm	550–50	296
Pr: TGG [107]	500–1300 nm	450–50	290
Terbium aluminum garnet (TAG) [108]	633–1064 nm	172–46	–
Pr: TAG [108]	633–1064	200–68	–
Ce: TAG [108]	633–1064	205–63	–
Cadmium manganese mercury telluride [3]	811–938 nm	1396–140	22.5–21
**Tb_2_O_3_** [109]	500–1500 nm	1000–50	20
Zinc telluride glasses (TeO_2_–ZnO–10.4Na_2_O, TeO_2_–ZnO–La_2_O_3_–Na_2_O) [110]	500–1500 nm	50–8	–
Holmium oxide (Ho_2_O_3_) [111]	633–1064 nm	178–46	–
Europium fluoride (EuF_2.1_) [112]	650–1075 nm	209–50	31

#### 2.2.1 Magneto-plasmonic metasurfaces

The combination of plasmonic resonances and magneto-optic effect, known as magneto-plasmonics, has been considered as a promising approach for developing nanoscale optical components. As we discussed in previous sections, magneto-optic effect for nonmagnetic materials is usually weak at optical frequencies. However, localized surface plasmon resonances in nanoscale plasmonic particles enhance the magneto-optic effect [30]. The plasma frequency, *ω*_p_, in metals is usually several orders of magnitude larger than cyclotron frequency, *ω*_c_ and if one considers a thin film of a plasmonic material, the magneto-optic effect is very weak for a feasible magnetic field bias. For nanoparticles, the localized surface plasmon resonance frequency is lower than plasma frequency in the bulk and the resonance frequency become closer to *ω*_c_, resulting in enhanced magneto-optic effects. Although plasmonic resonances increase the magneto-optic effect, such effects are still very weak in the absence of a magnetic material. A magnetic material enhances the magnetic field flux density $\left(B_{0}=\mu H_{0}\right)$ which results in a stronger magneto-optic effect. For instance, it has been shown that coating maghemite (γ-Fe2O3) nanoparticles with gold results in enhancement of Faraday rotation because of localized surface plasmon resonances in gold nanoparticles [113].

**Figure 2:**
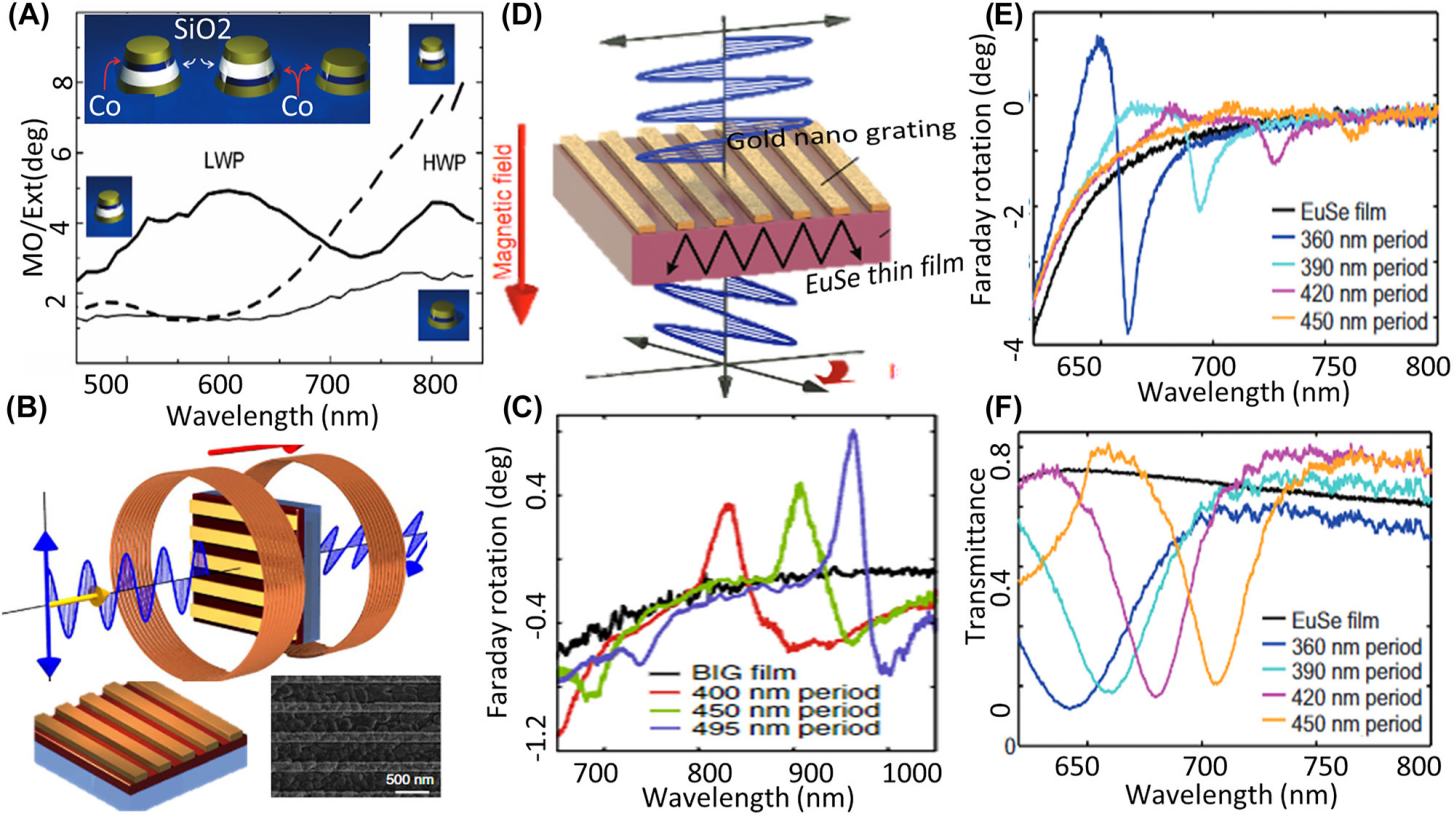
Enhancing Faraday rotation in plasmonic metasurfaces. (A) Array of gold/cobalt/gold nanostructures are studied for enhancing magneto optic effect. It is shown that adding a SiO_2_ layer between gold and cobalt results in reducing the loss and increasing the ratio of the magnetooptic effect to the extinction coefficient (adopted with permission from [115]). (B) and (C) gold nanogratings are created on top of the BIG thin film to create surface plasmon resonances and increase faraday rotation at visible and NIR frequencies [25]. (D) and (E) Gold nanogratings on top of the EuSe thin film instead of BIG are used for increasing the Faraday rotation [116].

Arrays of gold/cobalt/gold nanodisks have been studied as promising structures that can enhance the magneto-optic effect [24, 114]. Furthermore, to prevent loss in magnetic materials more complex structures containing several layers of metals, dielectrics and magnetic materials have also been used to redistribute the EM fields and reduce the absorption while enhancing the magneto-optic effect [115] as shown in Figure 2A. Although magneto-plasmonics has been studied for a decade and it has been suggested for controlling and modulation of surface plasmon polaritons [22, 23, 117, 118], achieving nonreciprocal transmission based on magneto-plasmonic metasurfaces is more challenging since both plasmonic materials and magnetic materials are lossy at optical frequencies. To have a considerable magnitude for the magneto-optic effect and high transmission at the same time, Belotelov et al., theoretically investigated adding a magnetic material to plasmonic metasurfaces showing extraordinary transmission [119]. Chin et al. fabricated and measured Faraday rotation from gold nanowires on top of a bismuth iron garnet (BIG) thin film, and they showed that the resonance in this structure enhances the Faraday rotation by a factor of 9 with respect to Faraday rotation from a bare thin film [25] as shown in Figure 2B and C. Furthermore, the same group utilized the same structure with europium selenide as the thin film and achieved larger Faraday rotation [29, 116] (Figure 2D–F).

#### 2.2.2 Magneto-optic effect in dielectric metasurfaces

**Figure 3:**
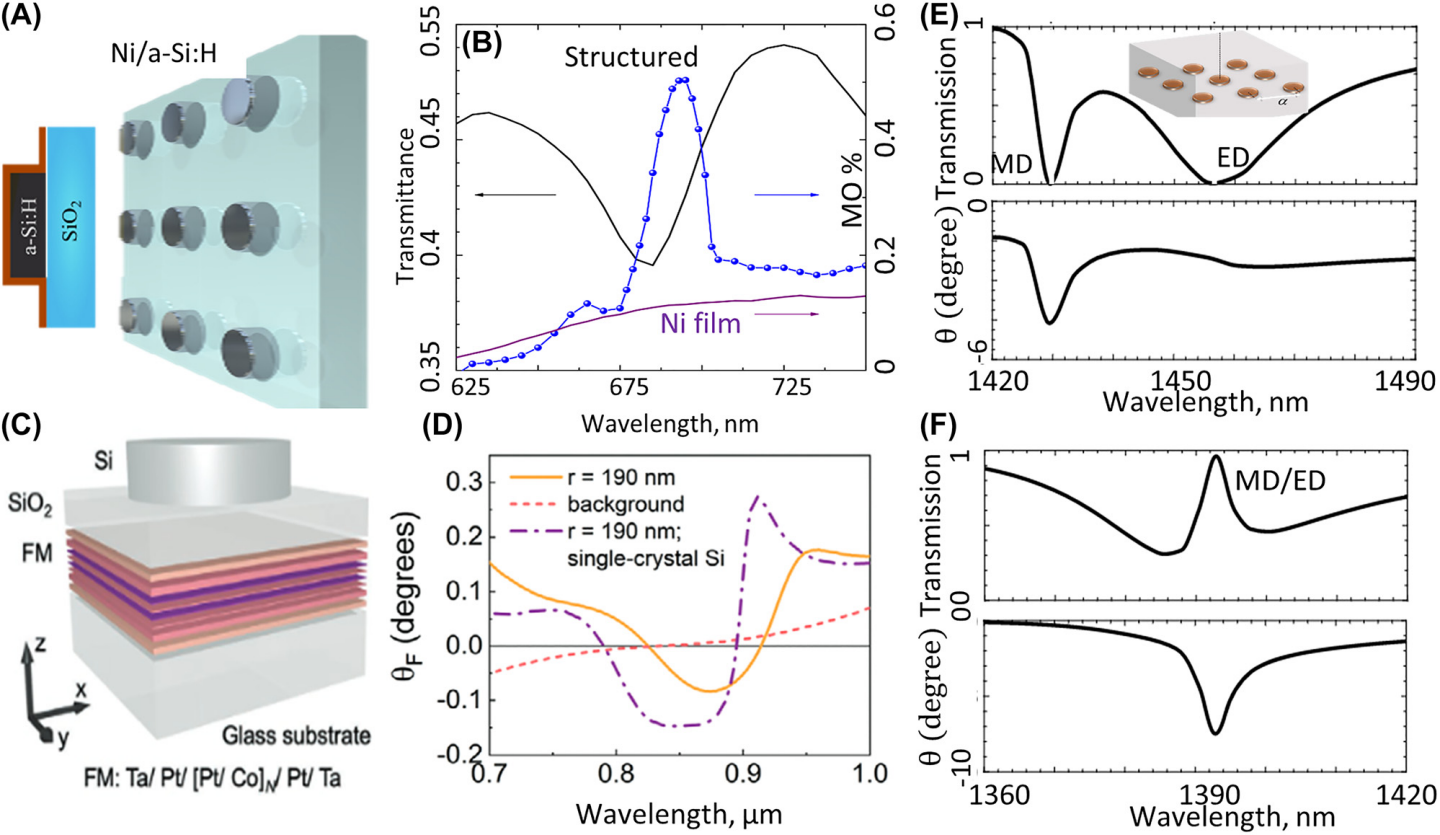
Enhancing Faraday rotation in dielectric metasurfaces. (A) and (B) Mie resonances in the a-Si:H cylinder that are covered with a 5 nm nickel layer and enhance the MO optic effect at visible wavelengths (adopted with permission from [32]). This figures shows that the peak of the Faraday rotation coincides with a deep in transmission. (C) and (D) Dielectric metasurfaces comprising Si nanodisks are fabricated on top of a thin film consist of multiple layers of platinum and cobalt stacked on top of each other. Faraday rotation for different nanodisk radius is measured to study the effect of Mie resonances on Faraday rotation (adopted with permission from [34]). (E) and (F) Simulation results for gold nanodisks that are suspended in a silica layer [33]. When electric dipole and magnetic dipoles are separated each resonance enhance the Faraday rotation but has negligible transmission as shown in (E). In (F) The radii of nanodisks are then adjusted to overlap electric dipole and magnetic dipole resonances and create high transmission while enhancing faraday rotation (adopted with permission from [33].

Although plasmonic metasurfaces provide extreme confinement of light on the subwavelength scale which enhances light–matter interactions, they suffer from loss at visible and NIR wavelengths. All-dielectric metasurfaces and metamaterials attracted a lot of attention over last decade because of their flexibility over plasmonic structures. Interaction between nanoparticles with EM fields can be described by multipole expansion of these structures. A similar approach to the one used in Section 2.2 can be used for explaining the effect of the magnetic field bias on the dielectric nanoparticles. Barsukova et al., experimentally studied the response of a metasurface comprising silicon nanodisks coated with nickel, and they demonstrate magnetic dipole resonances that enhance the magneto-optic effect [32] (Figure 3A and B). They also studied the enhancement of magneto-optic effect in the same structure when meta-atoms undergo electric dipole resonance [31]. Aside from using only one thin film of a magnetic material on top of dielectric metasurfaces, thin films involving a composite of different materials could also enhance the magneto-optic effect. Utilizing a thin film that consists of multiple layers of platinum and cobalt beneath a dielectric metasurface, Abendroth et al. showed enhancement of magneto-optic effect [34] (Figure 3C and D). Comparing magneto-optic enhancement in the vicinity of the magnetic dipole and electric dipole resonance frequencies revealed that magnetic dipole resonances have a more noticeable effect on the magneto-optic effect [31]. Magnetic dipole and electric dipole resonances can overlap with each other in a dielectric metasurface, also known as Huygens metasurfaces, which result in electromagnetically induced transparency (EIT). Christof et al. theoretically showed that one can achieve a considerable amount of Faraday rotation and close to unity transmission with structures showing EIT [33] (Figure 3E and F). Their proposed metasurface consists of bismuth-substituted yttrium nanodisks suspended in silica, and the metasurface is designed to have overlapping electric dipole and magnetic dipole Mie resonances. When electric dipole and magnetic dipole resonances do not have an overlap with each other, one can achieve enhancement in Faraday rotation with negligible transmission for each case. When two resonances overlap with each other, one can observe enhancement in Faraday rotation along with full transmission. Despite all the effort in achieving magneto-optic effect in all-dielectric metasurfaces, such structures can provide only limited AT.

One figure of merit that demonstrates how much magneto-optic effect has been enhanced in such structures is defined based on the difference between transmission for opposite directions of magnetic field bias

(4) $$\delta =\frac{T\left(H_{0}\right)-T\left(-H_{0}\right)}{T\left(H_{0}=0\right)}$$

where *H*_0_ represent the magnetic field bias. For all dielectric metasurfaces shown in this review that rely on magneto-optic effects, this figure of merit is less than 10%. Furthermore, Faraday rotation in magnetic materials can only lead to AT when they are used with polarizers, which further reduce the level of transmission.

#### 2.2.3 Graphene and transitional metal dichalcogenides

2-D materials and monolayers such as graphene with thickness on the order of one atom show distinctive optical and electronic properties that can be used for engineering very compact optical and electronic devices. The unique properties of 2-D materials also result in interesting behavior when they are exposed to a magnetic field bias. After demonstrating the intriguing electronic behavior of graphene in the presence of magnetic field bias and observing the integer quantum Hall effect in graphene [120], graphene’s behavior at higher frequencies drew a lot of attention to explore more information about its band diagram and investigate its potential application in optics. It has been shown theoretically that a magnetic field bias leads to a considerable Hall-conductivity – off diagonal component in the conductivity tensor – at terahertz frequencies, and it leads to Faraday rotation as EM waves pass through a monolayer of graphene [121, 122]. Indeed, in the presence of the magnetic field bias, the conductivity of the graphene can be described as:

$$\bar{\bar{\sigma }}=\left[\begin{array}{cc} \sigma_{\text{d}} & \sigma_{\text{o}}\\ -\sigma_{\text{o}} & \sigma_{\text{d}} \end{array}\right]$$

Similar to the procedure in Section 2.2 one can show that left-handed and right-handed circularly polarized light are eigenmodes for this surface conductivity with eigenvalues *σ*_cp_ = *σ*_d_ ± *σ*_o_ that are effective conductivity of the surface for two circular polarizations, and one can show that the amount of faraday rotation depends on the ratio between *σ*_o_ to *σ*_d_.

Faraday rotation through graphene is also investigated experimentally at low temperatures [123, 124]. In these experiments, the magnetic field bias varies from 0 to 7T and it was demonstrated that at frequencies close to 1 THz, graphene monolayer causes rotation of polarization angle in the order of 0.1 radians [123, 124]. After observation of the strong magneto-optic effect of graphene monolayer at terahertz frequencies, various methods have been suggested in the literature for structuring the graphene sheets to enhance the interaction of graphene with EM waves and improve the Faraday rotation in graphene [125–129]. For example, magneto-plasmonic resonances in graphene microribbons are investigated, and it was shown experimentally that arrays of microribbons enhance the Faraday rotation at higher frequencies [126]. More recently, the combination of graphene with gold nanogratings on top of the silicon hybrid slabs has been used to theoretically show strong Faraday rotation up to 24 THz [127]. Due to its unique properties, graphene could be a promising candidate for providing ultrathin optoelectronic devices, but there are various challenges for providing the required magnetic field bias and temperatures for realizing devices based on graphene, as discussed in [130].

Recently, transition metal dichalcogenides (TMD) [131], another important class of 2-D materials, have been used for breaking reciprocity in the presence of an electric field pump [132]. In crystal structures with multiple lattice sites, degenerate energy levels can exist such that the minimum of the conduction band and the maximum of valence band with the same band gap occur at two separate points of the Brillouin zone. It has been shown that in TMD crystals such as molybdenum disulfide (MoS_2_) and Tungsten disulfide (WS_2_) two valleys with the same band gap exist such that inter-band transition of electron (hole) at two valleys results in excitations with distinct circular polarization direction [133–136]. This phenomenon is considered as a promising approach for creating optoelectronic systems in TDM by controlling the polarization of excitation. Recently, valley-polarization property of TDM has been used for breaking reciprocity in the presence of an electric field pump [132]. When excited with a strong circularly polarized laser, the density of excited electrons that move to the conduction band is higher at one of the valleys compared to the other one. Since two valleys have distinct momentum (at points K and K′ in the reciprocal state), the circularly polarized excitation induces an asymmetry in the conductivity tensor of the 2D material, which breaks the reciprocity. It is discussed that laser pulses with the energy of 450 µW are enough to create a nonreciprocal reflection response. An all-optical isolator at 615 nm is then proposed based on this behavior that creates 20 dB isolation at this wavelength. One should notice that the reciprocal response in this system relies on the carrier generation with the circularly polarized light, and the pulse width and frequency of the laser pulses affect the response.

Applying static and dynamic bias fields is therefore a promising approach for achieving nonreciprocal responses from 2-D materials.

### 2.3 Breaking reciprocity in nonlinear asymmetric structures

Using a magnetic field bias and magnetic materials is not desirable for various applications where we need to integrate several components in a very small area. Instead of applying a magnetic field bias, one can use nonlinear materials to create intensity-dependent refractive index for breaking the reciprocity. The basic principle for breaking reciprocity with nonlinear materials is to create different permittivity distributions for light propagating along opposite directions [22]. One can show that in a structure lacking mirror symmetry, the intensity of the electric field at a fixed point can be different when the structure is excited from opposite sides. Assuming the intensity of the electric field is high enough to create a considerable intensity-dependent refractive index, the asymmetry in the field profile leads to a different effective index for waves propagating in opposite directions, which results in breaking the reciprocity. Such structures are also known as self-bias nonreciprocal structures.

**Figure 4:**
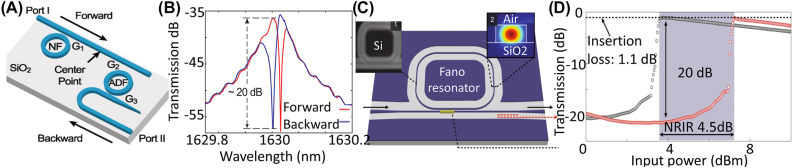
On-chip AT based on nonlinear resonators. (A) Shows the schematic of the on-chip isolator using a nonlinear resonator coupled with waveguides along with the frequency filter, and (B) shows the transmission response for forward and backward directions (adopted with permission from [36]). (C) Demonstrates the on-chip isolator based on inversed designed Fano resonator designed for a nonreciprocal pulse router and (D) shows forward and backward transmission with respect to the input power (adopted with permission from [37]).

This principle has been used for photonic circuits to create optical diodes that block the back reflection of high-intensity signals. In [23], an asymmetric photonic circuit comprising a silicon microring resonator and silicon waveguides, Figure 4A, is designed so that forward and backward signals have distinct coupling values to the resonator. Different coupling efficiencies to the resonator result in dissimilar levels of stored energy in the micro ring resonator and because of the nonlinear properties of silicon, the resonance frequency varies when excited from different ports resulting in 20 dB isolation as shown in Figure 4B.1One should note that isolation is defined as the ratio between forward transmission to backward transmission and an ideal isolator has infinite isolation which means zero transmission for the backward direction [137]. Here we are showing the isolation level when a specific isolator is shown. In such structures, using a resonator with a Fano-type resonance provides more isolation than Lorentzian resonators. The asymmetric spectrum of Fano resonance is preferred since with a slight shift in the frequency, transmission level can change from a peak value to a minimum, which is desirable when we are looking for AT through nonlinear effects and frequency detuning. Yang et al. utilized a nonlinear asymmetric Fano resonator in a photonic circuit, Figure 4C and D, and achieved isolators with very low insertion loss [98]. In nonreciprocal structures that operate based on the nonlinear effect, the isolation level between forward and backward transmission depends on the intensity of the excitation from opposite directions, and such structures are evaluated assuming the same intensity for forward and backward excitation. In isolators that are realized based on coupling between a nonlinear resonator to two input and output ports, it has been shown that using coupled mode theory one can extract a relation for forward and backward transmission. If one considers the forward and backward input lights, say *I*, this theoretical model shows that as *I* varies, there is a sharp transition in forward transmission from a low value to a high value, happens at *I*_1_, followed by a sharp transition back to a low value, happens at *I*_2_. Assuming that backward transmission is negligible, the system shows a considerable amount of isolation for intensities ranging from *I*_1_ to *I*_2_ [138]. The ratio between these two intensities where there is a fast transition in the transmission level is known as the nonreciprocal intensity range (NRIR). Based on the model developed from coupled mode theory, it has been shown that there is a trade-off between the maximum forward transmission level, *T* ^f^, and the range of intensity for which there is a considerable isolation level [138]:

(5) $$T^{\text{f}}\leq \frac{4\text{NRIR}}{{\left(\text{NRIR}+1\right)}^{2}}$$

The relation for forward transmission in (5) is valid independent from the backward transmission and is, therefore, only an upper limit for the isolation level. However, one should note that in such isolators that operate based on the nonlinear resonator, the transmission is negligible if the resonator is not at resonance, which means that the system is designed to have a negligible backward transmission at the desired frequency, and the resonator can only be at resonance for forward excitation. Therefore, this relation shows a tradeoff between the insertion loss in the system and the range of input powers that show nonreciprocal transmission. Table 3 represents some of the power ranges and the isolation levels measured in isolators that are based on nonlinear materials. It has been demonstrated that increasing the number of resonators can break the limitation between nonreciprocal intensity range and maximum level of transmission [40].

**Table 3:** Range of incident powers and frequencies for on-chip and waveguide isolators based on nonlinear materials.

Pump laser power	Wavelength	Isolation level (dB) (the ratio between	Nonlinear resonator
		forward and backward transmission in dB)	
5–15 dBm [36]	1630 nm	22–28 dBm	Silicon micro ring
4–9 dBm [37]	1550 nm	20–15 dBm	Silicon micro ring
5–25 dBm [139]	1550 nm	10–30 dBm	Fused silica microrod resonator coupled to fiber
30 dBm [35]	Microwave (not reported exactly)	10 dBm	Varactor

Sounas et al. experimentally showed for microwave frequencies that the combination of a nonlinear Fano resonator and a nonlinear Lorentzian resonator widens acceptable power ranges that create nonreciprocal transmission.

The idea of coupling nonlinear resonators has also been investigated in photonic crystal waveguides with different defects [38, 140, 141], since photonic crystals provide very good control over the coupling between a photonic crystal waveguide and defects that acts as resonators. However, such structures are usually challenging to fabricate.

Using multiple resonators in order to enhance the power range for nonreciprocal response led to complex structures and enhancing the insertion loss which reduces the efficiency of nonlinear nonreciprocal systems. Adding a gain medium to the system could compensate for the loss, and it provides the possibility of cascading multiple isolators in relation to each other.

Indeed, a combination of resonators with loss and gain media results in parity-time (PT) symmetric systems which demonstrate many interesting behaviors including AT [142–144]. For example, Peng et al. demonstrated that coupling a nonlinear resonator with a gain medium and a lossy nonlinear resonator result in nonreciprocal transmission and showed that the required range of the input powers in their structure is lower than other structures without gain [145].

#### 2.3.1 Nonlinear materials in asymmetric metasurfaces

**Figure 5:**
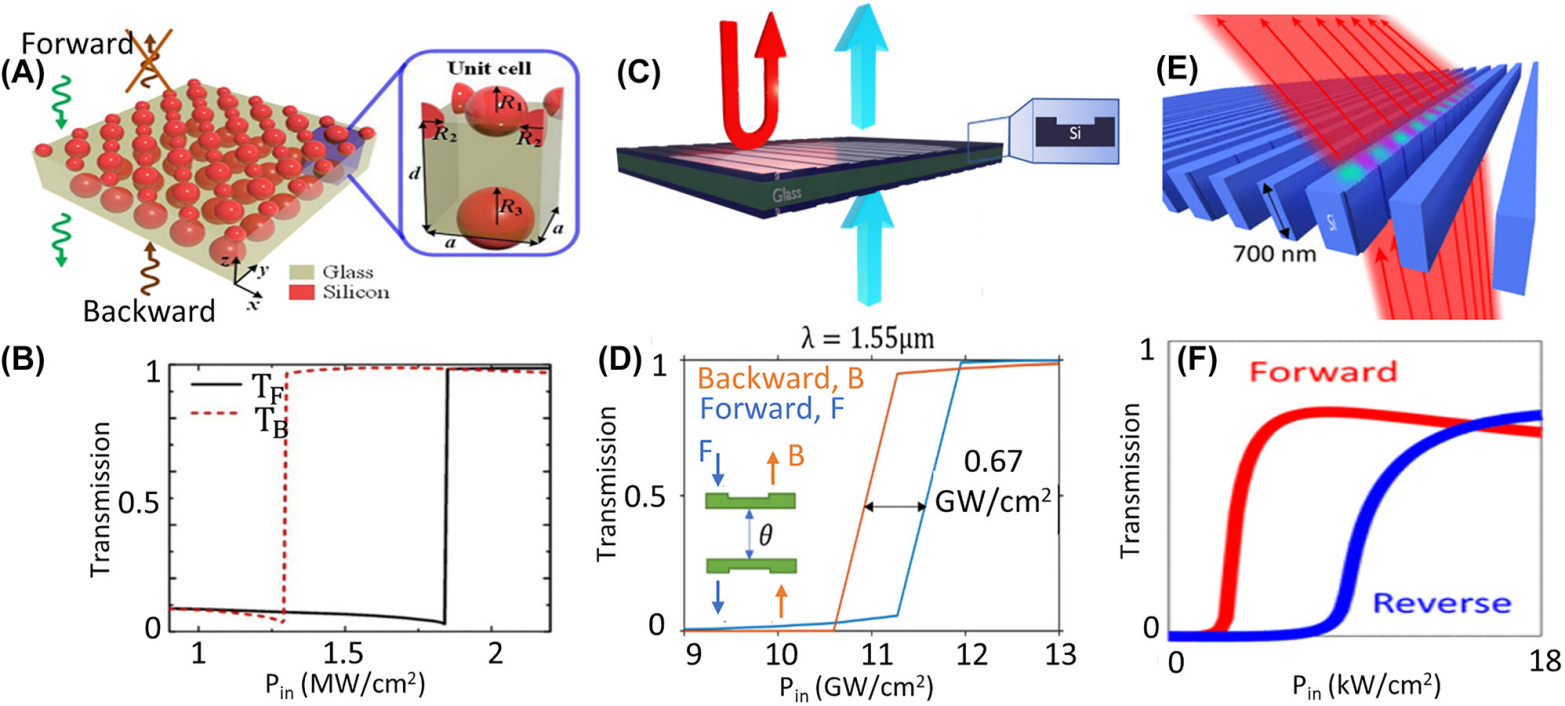
Demonstration of intensity dependent AT metasurfaces with simulations and theoretical models. (A) and (B) Si nanospheres are arranged on two sides of glass such that on one side they show a Fano type resonance, and on the other side they show a Lorentzian resonance. Coupled nonlinear Fano resonator and Lorentzian resonator results in AT for *λ* = 1530 nm (adopted with permission from [43]). (C) and (D) silicon gratings on two sides of a silica layer are designed to create coupled nonlinear Fano that provide AT for 1550 nm [42]. (E) and (F) nanogrooves on side walls of an array of silicon bars with gradient phase for nonreciprocal beam steering at 700 nm (adopted with permission from [41]).

Aside from waveguide structures and photonic crystals, coupled nonlinear resonators have also been investigated in metasurfaces. In contrast to integrated photonic circuits where light is confined to waveguides and microrings, an optical metasurface is usually excited with a free space beam with beam spots ranging from a few to tens of micrometers. Thus, achieving nonlinear effects and controlling the coupling between different elements is more challenging in metasurfaces. Until now, all of the research related to demonstrating AT in a nonlinear metasurface has been restricted to theoretical investigation and simulations. Asymmetric double layer metasurfaces where two layers show nonlinear resonances can effectively behave like two nonlinear on-chip resonators that couple to a waveguide. Jin et al. proposed asymmetric layers of silicon nanosphere arrays, which are separated with a thin glass for creating nonlinear nonreciprocal transmission [43], Figure 5A and B. The authors use two spheres with different radii in each unit cell to have a nonlinear Fano-type resonance in one of the layers. Metasurfaces based on nonlinear Fano-resonances in double-layer silicon gratings have also been analyzed theoretically for creating nonreciprocal transmission, Figure 5C and D [42]. In [41], a nonreciprocal beam steering metasurface is proposed and it has been shown that adding arrays of asymmetric grooves in a silicon slab, Figure 5E, can create nonreciprocal transmission for incident powers ranging between 2 kW/cm^2^ to 10 kW/cm^2^ (Figure 5F). Arranging grooved slabs with different thicknesses is then investigated for creating beam steering while having a nonreciprocal transmission.

The nonreciprocal nonlinear structures (both waveguide structures and metasurfaces) discussed so far rely on the nonlinear Kerr effect which can be modeled with third order susceptibility, *χ* ^(3)^, or an equivalent nonlinear refractive index. Most nonreciprocal structures experimentally realized so far use silicon for creating a nonlinear behavior since it presents considerable nonlinear properties at communication wavelengths, and we have extensive knowledge and techniques for its fabrication. However, other semiconductors such as GaAs or chalcogenide glasses such as AsSe also show high nonlinear refractive index, *n*_2_, which can be used for designing nonreciprocal transmission. Table 4 shows refractive index and nonlinear refractive index2When nonlinear optical properties of the material arise from *χ* ^3^, it is common to represent the total effective refractive index as a linear term and a nonlinear term as: *n* = *n*_0_ + *n*_2_ *I* where *I* represents the intensity. of various materials at 1550 nm.

**Table 4:** Nonlinear refractive index and refractive index of common dielectrics with considerable nonlinear kerr effect at 1550 nm.

Material	*n*_0_	*n*_2_ × 10^−18^ (W^−1^ m^2^)
Si	3.5	4
AlGaAs	3.3	26
GaP	3.1	11
SiN	2	0.25
SiO_2_	1.4	0.022
**AS_2_S_3_**	2.9	–
**As_2_Se_3_**	12	12
GaAs	3.4	15.9

#### 2.3.2 Volatile materials in asymmetric metasurfaces

Instead of relying on optical Kerr effect to create a refractive index that varies with intensity, one can use volatile phase change materials such as vanadium dioxide (VO2), for which the permittivity can be controlled with temperature. VO2 experiences metal-to-insulator transition at specific threshold temperatures [146–148] and it shows different optical properties across the two phases.

An optical beam with high intensity can considerably increase the temperature in judiciously designed VO2 thin films to change the VO2 from insulator to a metal. Based on this principle, one can design asymmetric metasurface containing VO2 thin films such that for excitation from one side the incident light leads to an insulator to metal transition and results in negligible transmission while for excitation from the opposite side VO2 remains as an insulator with higher optical transmission. Wan et al. demonstrated AT based on the phase change properties of VO2 [44] where a combination of gold and VO2 thin films on a sapphire substrate was used. This architecture creates asymmetric field intensity for backward and forward excitations, and it was shown that a 40 mW continuous-wave laser source causes an insulator-to-metal transition and zero transmission for excitation from one side while excitation from the opposite side with the same power does not change the insulator VO2 to a metal and shows higher levels of transmission.

#### 2.3.3 Nonlinear optomechanical effect in asymmetric structures

**Figure 6:**
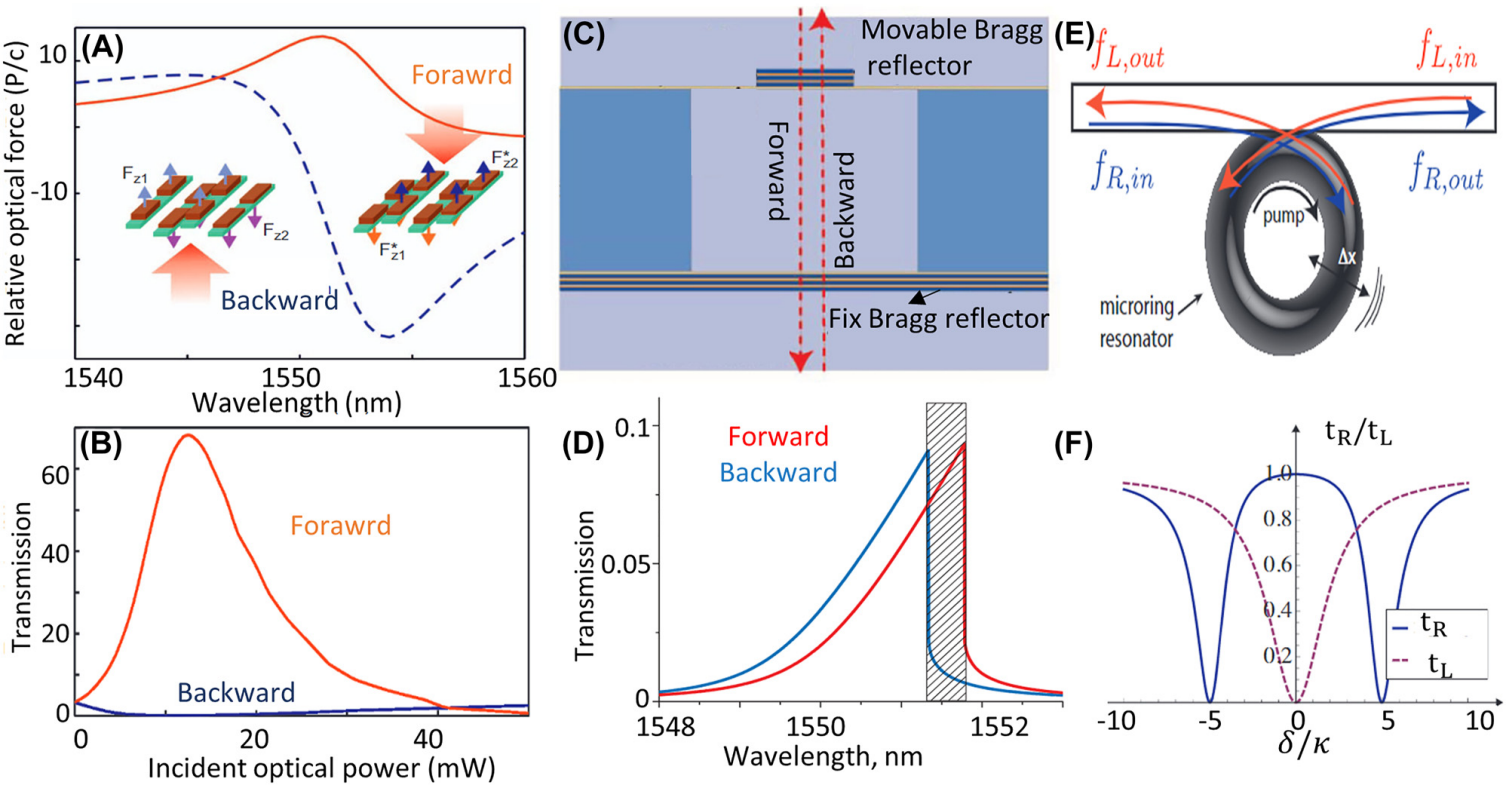
Intensity dependent AT based on optomechanical effect. (A) and (B) AT based on opto-mechanical force on Si nanobeam fabricated on silicon nitride membrane for input power around 0.2 mW/µm^2^ at 1550 nm [47]. (C) and (D) AT from a resonator formed between a fixed and a movable Bragg reflector created using multiple layers of Si and SiO_2_ when incident power is around 100 mW/µm^2^ (adopted with permission from [49]). (E) and (F) Schematics and simulation results showing how the optomechanical coupling between mechanical modes and optical modes of a ring resonator led to nonreciprocal transmission [149]). *t*_L_ and *t*_R_ represents the transmission of waves to left and right, *δ* is detuning from the resonance frequency of resonator and *κ* represents the coupling between waveguide and resonator (adopted with permission from [149]).

Another approach for creating intensity-dependent effective index is to use optomechanical systems. In optomechanical systems, high intensity light can cause considerable mechanical movements that affect the overall transmission. Similar to nonlinear structures, one can design asymmetric structures possessing optomechanical couplings to create different effective indices for the light propagating in opposite directions. Zhang et al. demonstrated that optomechanical resonances in silicon nanobars deposited on a silicon nitride membrane, Figure 6A and B, can create nonreciprocal transmission because the movement of nanobars is different for excitation from opposite sides [47]. Nonlinear optomechanical effects have also been reported in cantilevers where the asymmetric intensity distribution of light on the cantilever creates asymmetric force on the cantilever and results in AT [46].

One straightforward approach for creating significant optomechanical effects is to use a fixed reflector and a mobile reflector to form a tunable cavity. In such a structure, multiple reflections from two reflectors forms a resonance, and the optical force on the mobile reflector can move the reflector and create optomechanical coupling. Manipatruni et al. showed nonreciprocal transmission by designing a cavity formed by two Bragg reflectors where one of the reflectors was designed was based on a cantilever to have mechanical movements [49], Figure 6C and D. It was shown that the optical force on the mobile Bragg reflector is different for excitation from opposite directions and the asymmetric optical force results in AT. Another scenario where optomechanical effects lead to nonreciprocal transmission is to use an additional pump signal for excitation of mechanical resonances. In systems possessing optomechanical coupling, an optical signal can create a mechanical bias which breaks the time-reversal symmetry and creates nonreciprocal transmission. Such systems can be realized using a silica microtoroid optomechanical resonator [150] where optical signals can excite mechanical modes in the resonator, as shown in Figure 6E. When an optomechanical resonator is excited with a pump signal, a mechanical eigenmode is excited in the resonator. The mechanical eigenmode could have constructive interference with the wave propagating in the forward direction in the coupled waveguide, achieving a high level of forward transmission. However, the wave propagating in the opposite direction destructively interferes with the resonator and has negligible transmission [149], Figure 6E and F. Ruesink et al. experimentally demonstrated this idea utilizing silica microtoroid optomechanical resonators [48].

Like other methods mentioned in this section, AT based on optomechanical effects depends on the incident beam intensity. Besides, implementing coupled optomechanical systems requires accurate control over the resonance frequency and coupling between the resonator and the waveguide, which needs to be monitored and tuned externally. Additionally, the fabrication and integration of such systems with other optical components is challenging.

#### 2.3.4 Dynamic nonreciprocity limitation

Another limitation when using the intensity of the electric field for creating AT is that such structures cannot operate effectively when they are excited simultaneously from both directions [37]. In these structures, excitation from one direction creates very intense fields that change the structure’s effective index. If we imagine a scenario where a structure is simultaneously excited with continuous laser sources from both directions, the effective index of the medium for waves propagating in opposite directions is the same, disabling any AT effects.

### 2.4 Spatiotemporal modulation

#### 2.4.1 Principles of breaking the reciprocity based on spatiotemporal modulation

In a static lossless medium where the permittivity distribution is not changing with time, time-reversal symmetry is equivalent to reciprocity since the action of the time-reversal operator on the system is equivalent to changing the direction of the propagation. In a time-varying medium, applying the time-reversal operator on the system changes the properties of the system, and the relation between time-reversal symmetry and nonreciprocity is more complex.

Consider a nonreciprocal system in a medium involving time modulation; in such systems, we expect the EM waves propagating in opposite directions to show a different behavior while permittivity distribution has the same time modulation for both forward and backward waves. However, applying the time-reversal operator to such a time-varying medium changes both the permittivity distribution and the direction of propagation. Therefore, in a system that involves time-modulation, breaking the time-reversal symmetry does not necessarily break the reciprocity. For example, if we consider a waveguide whose permittivity at a specific point is changing sinuously as *ɛ* = *ɛ*_*m*_ sin(*ω*_*m*_ *t*), the transmission in this waveguide remains symmetric as can be shown from Lorentz reciprocity theorem in the time domain.

Even though adding simple time-modulation in a system cannot create nonreciprocal transmission, spatiotemporal modulation in a system can break the reciprocity. The simplest form of a spatiotemporal modulation, is traveling-wave modulation where the permittivity distribution is described as:

(6) $$\varepsilon =\varepsilon_{b}+\varepsilon_{m}\sin\left(\omega_{m}t-\beta_{m}x\right)$$

Such permittivity distribution has been used in microwave devices for parametric amplification [151]. One can think of travelling-wave modulation as a permittivity distribution moving with velocity $v=\frac{\omega_{m}}{\beta_{m}}$ . If we consider the propagation of forward and backward waves in such a medium, the relative velocity of the forward and backward wave with respect to the moving medium is different with each other (Doppler effect) and the moving permittivity creates a nonreciprocal response.

To have a more accurate explanation for the behavior of an EM wave with spatiotemporal modulation, let us consider the dispersion relation of waves propagating in the bulk of such a medium [152]. We assume the medium is uniform along the *x* and *y* direction and it has a traveling-wave modulation occurring along the *z* direction as $\varepsilon \left(z,t\right)=\varepsilon_{b}+\varepsilon_{m}\sin\left(\omega_{m}t-\beta_{m}z\right)$ .

A wave that is propagating along the *z* direction in such medium is a solution of the following wave equation, for simplicity we assume electric field has a linear polarization along the *y* direction, and we neglect variation along the *x* direction (2-D problem). Since the structure is periodic along *z* and *t*, according to the Floquet–Bloch theorem the solution can be written as:

(7) $$E=\sum E_{n}{\text{e}}^{\text{i}\omega_{0}t-\text{i}\beta_{0}z-k_{y}y}{\text{e}}^{-in\left(\beta_{m}z-\omega_{m}t\right)}$$

Substituting (7) in Maxwell equations results in a system of linear equations and setting the determinant of the coefficient matrix equal to zero results in the dispersion relation between *ω*, *β*, *k*_*y*_. The travelling-wave modulation introduces an asymmetry for the dispersion of EM waves propagating in +*z* (with wavenumber $\beta_{0}^{+}$ ) and −*z* (with wavenumber $\beta_{0}^{-}$ ).

When the amplitude of modulation is very small, *ɛ*_*m*_ ≪ *ɛ*_*b*_, it has been shown that the dispersion relation can be simplified as [152]:

(8) $$k_{y}^{2}\pm {\left(\beta_{0}^{\pm }\pm n\beta_{m}\right)}^{2}={\left(\frac{\omega_{0}+n\omega_{m}}{v_{b}}\right)}^{2},\;v_{b}=\frac{c}{\sqrt{\varepsilon_{b}}}$$

Spatiotemporal modulation creates periodicity in space and time which results in space-time harmonics. *nβ*_*m*_ and *nω*_*m*_ in (8) represents these harmonics. Considering two waves propagating along the *z* direction, *k*_*x*_ = 0, one can see that for two waves propagating along *z* and -*z* have different propagation constants $\beta_{0}^{+}$ and $\beta_{0}^{-}$ .

#### 2.4.2 Spatiotemporal modulation in waveguides and metasurfaces

The arguments mentioned so far are valid for infinite media. For a practical scenario such as an optical waveguide where only discrete eigenmodes can propagate in the structure, achieving nonreciprocity by applying spatiotemporal modulation is more challenging. Adding spatiotemporal modulation in a waveguide modulates the frequency and the momentum of optical modes, and frequency and phase matching are necessary for creating propagating modulated waves.

In 2009 Yu et al. theoretically demonstrated that nonreciprocal transmission based on spatiotemporal modulation in a waveguide is possible through an indirect interband photonic transition [58]. It was demonstrated that one can design spatiotemporal modulation in an optical waveguide such that in a perfect phase matching condition, the energy of a wave propagating in a specific direction can completely be transferred to a higher mode of the waveguide. However, the phase matching (or momentum conservation) condition is not valid for the wave propagating in the opposite direction and this mechanism can be used for creating nonreciprocal transmission.

**Figure 7:**
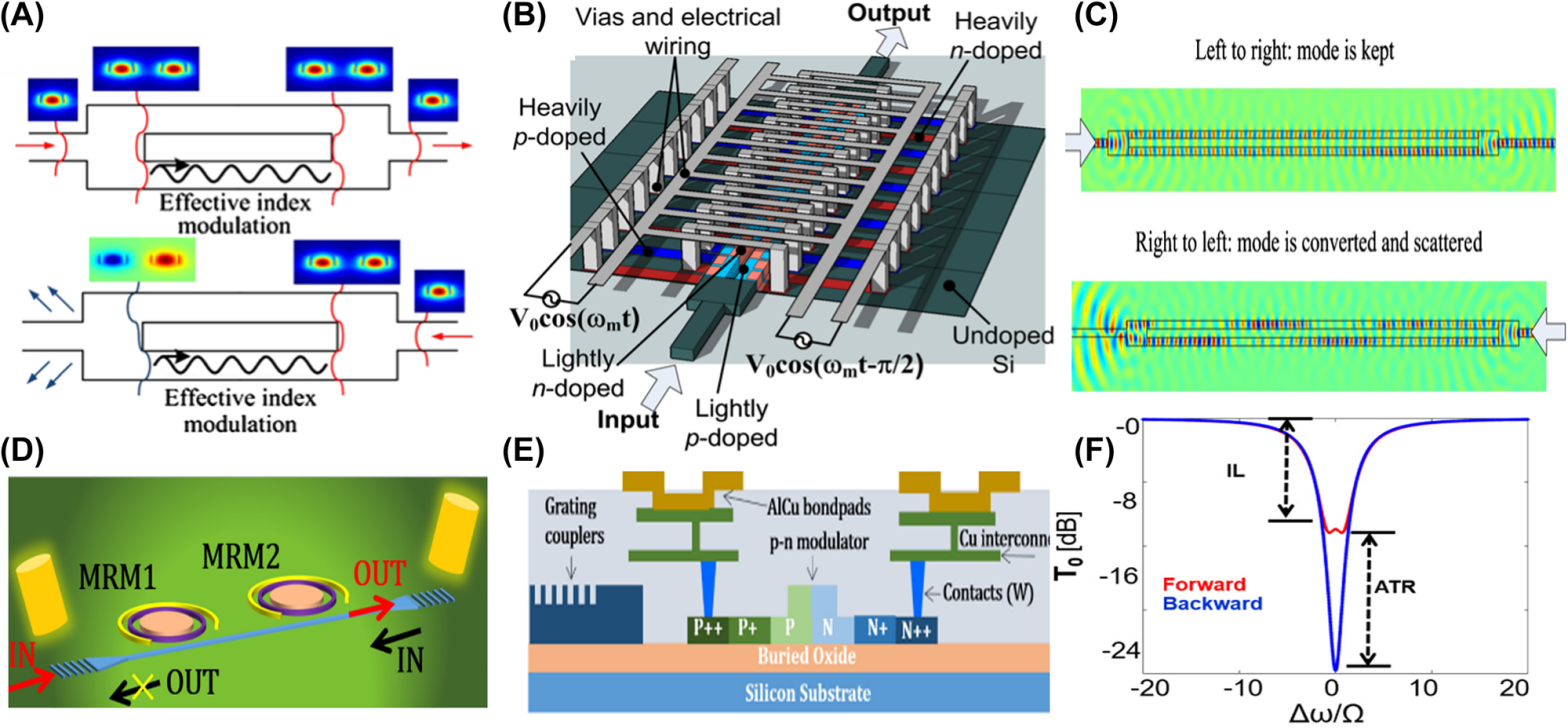
Nonreciprocal transmission based on travelling-wave modulation in waveguide structure. (A) shows the schematic of two waveguides that are coupled through a medium with spatiotemporal modulation, and the mode transmission in different directions happens for only one direction resulting in AT [51]. (B) Shows the model for realizing the system in (A) where modulating the pn junctions along a transmission line creates the required spatiotemporal modulation [51]. (C) Shows simulation results for this system (A–C are adopted with permission from [51]). (D) shows schematic of the structure proposed for nonreciprocal transmission based optical ring resonators having time modulations with proper phase delay between two modulations to create a nonreciprocal response [53]. (E) shows the cross section of the structure and (F) shows simulation results [53]. Measurement results for this system shows 16 dB isolation at 1560 nm (adopted with permission from [53]).

The main challenge in these structures is that available modulation frequencies are orders of magnitude smaller than communication wavelengths which results in large footprints in these structures. To achieve transition between optical modes using a small frequency modulation, nonreciprocal transmission based on interband optical transition between two spectrally close even and odd modes was also suggested [51], Figure 7A–C. In this work, the authors theoretically investigated a structure comprising coupled silicon waveguides supporting two modes at two frequencies separated by a few GHz, and they realized spatiotemporal modulation by using pn junctions connected to microwave transmission lines where the travelling-wave along the transmission line results in travelling-wave modulation of carriers in the photonic waveguide.

Utilizing the same principle, an experimental realization of an on-chip isolator was proposed using time modulation in cascaded silicon microring resonators instead of wave guides to reduce the footprint of the on-chip isolators which demonstrated 16 dB difference between forward and backward transmission [53], Figure 7D–F.

Since available modulation frequencies are in the order of tens of gigahertz, most demonstrations for nonreciprocal structures based on spatiotemporal modulation have been done across microwave frequencies, where modulation frequencies are on the order of the energy difference between two modes [63, 66, 68, 153], [154], [155], [156]. Indeed, for microwaves the idea of implementing spatiotemporal modulation for achieving nonreciprocal transmission goes back to 1960 [157], when it was shown that two varactors that are modulated with two pump signals with 90-degree phase shift and are connected with a quarter-wave transmission line produce nonreciprocal transmission. This idea has been generalized to create nonreciprocal transmission lines based on varactors [158]. Although using varactors in a transmission line allows transmission only in one direction, the frequency of the input and output signals is different which is not desirable in various applications.

Another approach suggested for using varactors is to create an angular modulation bias around a ring [55, 159]. In such structures, spatiotemporal modulation is created along a ring that is coupled to input and output ports. The modulation in the ring splits degenerate left-handed and right-handed modes in the ring. This creates asymmetric coupling of forward and backward waves and therefore AT. Since varactors can provide limited depth of modulation ( $\frac{\varepsilon_{m}}{\varepsilon_{b}}$ in Eqs. (1) and (10)), switching network based on CMOS transistors has been proposed for creating conductivity modulation to achieve nonreciprocal devices in the microwave [160–163]. Such switches are suitable for RF frequencies, but it is challenging to increase the operational frequency in these devices.

In a metasurface the thickness is subwavelength, and we cannot use the propagation of waves along the thickness of the metasurface. Instead, we can use the propagation of leaky waves along the surface of a metasurface. Spatiotemporal modulation in the form of travelling-wave modulation along the surface of a metasurface breaks the reciprocity and results in AT.

For example, Shi et al. theoretically demonstrated that electro-optic modulators can be used for creating spatiotemporal modulations in photonic crystal slabs [59]. It has been shown that the photonic crystal slab supports multiple modes whose eigen frequencies are close to each other, such that adding relatively small modulations in these structures leads to nonreciprocal transitions between different modes. In this work, it has been assumed that we can use electro-optic modulators to modulate the permittivity of each unit cell with a desirable phase shift among modulators such that the permittivity distribution can be represented as $\varepsilon \left(x,y,t\right)=\varepsilon_{s}\left(x,y\right)+\varepsilon_{m}\left(x,y\right)\cos\left(\Omega t+\phi \left(x,y\right)\right)$ .

For such a modulation to create a transition between two modes in the photonic crystal slab, the phase of the modulator at each point, $\phi \left(x,y\right)$ , is designed in a way that its spatial derivative compensates for the momentum mismatch between two modes of the photonic crystal, $\nabla \phi \left(x,y\right)=\Delta \vec{K}$ .

**Figure 8:**
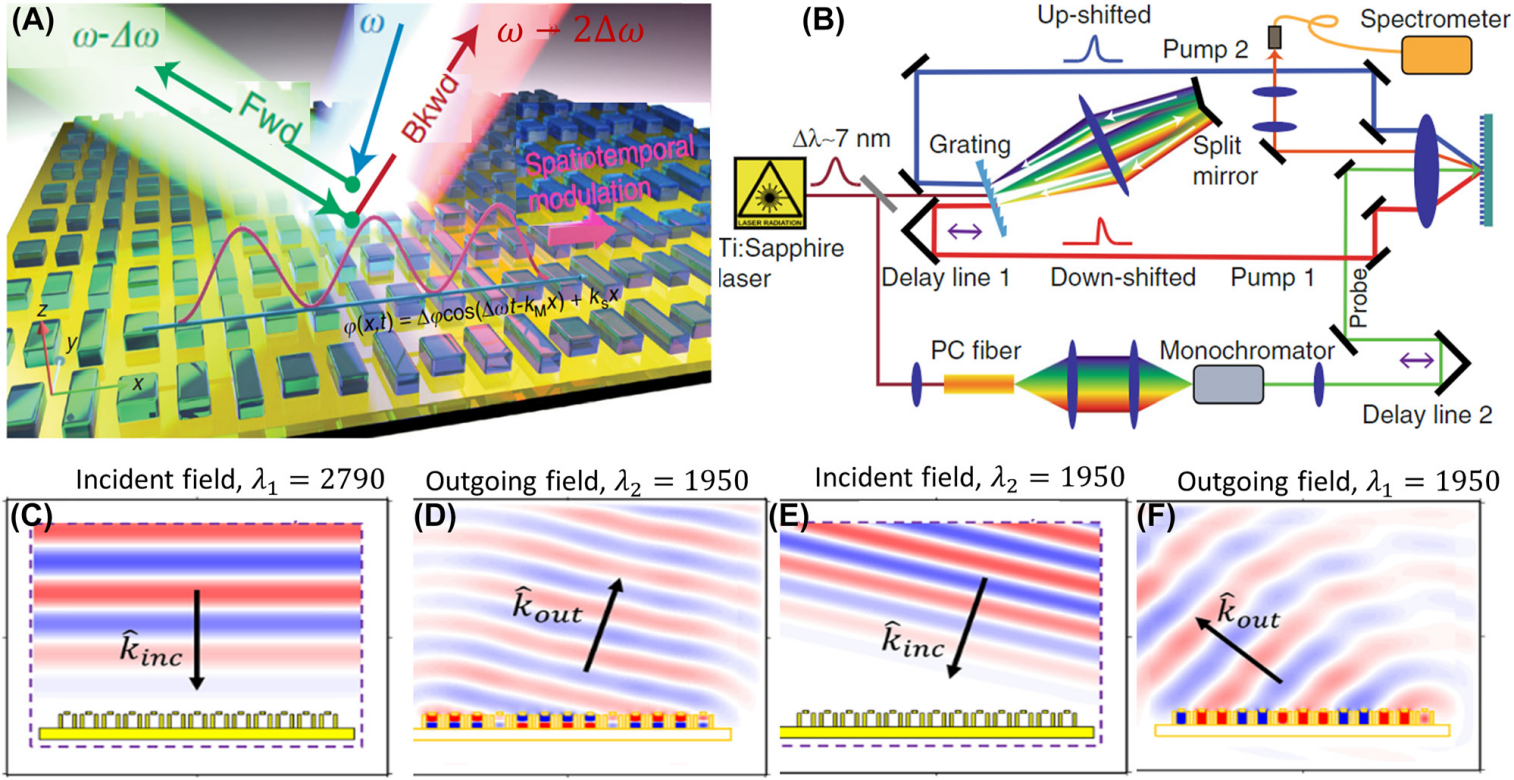
Spatiotemporal modulation in metasurfaces. (A) and (B) shows the metasurface and the setup used for creating spatiotemporal modulation using two high intensity laser sources to transfer the spatiotemporal modulated intensity profile to nonlinear meta molecules [62]. (C)–(F) simulation results of the metasurface demonstrating nonreciprocal behavior. The normal excitation scatters the light to *k*_out_, *λ*_2_ state as shown in (C), and (D). However, exciting the metasurface with those frequency and momentum does not lead to the initial incident wave as shown in (E), and (F) (adopted with permission from [60]).

Guo et al. experimentally demonstrated a nonreciprocal metasurface by implementing a spatiotemporal metasurface based on high-intensity laser sources [62], Figure 8A andB. In this work, the authors used two laser sources to create a heterodyne interference pattern which can create a traveling wave intensity distribution. Furthermore, they adjusted the intensity of the two laser sources to be high enough to use nonlinear properties in silicon meta-atoms in the structure. Therefore, they transferred the input travelling-wave intensity distribution from two lasers to a spatiotemporal modulated metasurface. The complex setup and optical bias in this work restrict its application for compact integrated optics and photonic systems. At microwave frequencies, the unit cells have larger sizes, and the modulation frequencies are on the order of the operational frequency, as such nonreciprocal metasurfaces based on spatiotemporal modulation are mostly realized in microwave frequencies.

Instead of a travelling-wave modulation along the surface and creating a phase shift between individual meta-atoms, it has been proposed that a nonreciprocal metasurface can be designed by applying a time modulation with a fixed phase for all unit cells in a spatially gradient metasurface [60]; the permittivity in such metasurfaces can be described as $\varepsilon =\varepsilon_{s}\left(x,y\right)+\varepsilon_{m}\left(x,y\right)\cos\left(\Omega t+\phi_{0}\right)$ .

This principle has been used in a phase gradient metasurface supporting two modes, and based on the coupled mode analysis, it has been shown that adding time modulation to all the unit cells of a phase gradient metasurface results in a nonreciprocal response [60], Figure. 8C–F. Similar response has been demonstrated theoretically by FDTD simulation of time-varying gradient metasurfaces [164]. We should note that in theoretical models developed for the analysis of spatiotemporal metasurfaces, it is assumed that the time modulation is slow enough that one can assume the permittivity at each time only depends on the electric field in that time and not the electric fields at previous times. For fast modulation schemes, this assumption is not valid, and more complex considerations are required.

#### 2.4.3 Realizing spatiotemporal modulation

One of the challenges in achieving a nonreciprocal response based on spatiotemporal modulation is choosing the appropriate material for obtaining the required time modulation. Generally, any material whose optical properties can be changed by applying an external energy could be used for creating the desired modulation in such structures. Modulating carrier concentration in silicon, utilizing the second-order nonlinearity in materials such as lithium niobate, barium titanate (BTO) or lead zirconium titanate, and 2D materials such as graphene are examples of materials that have been used for achieving time modulation [69]. Phase change materials such as chalcogenides are an alternative approach for realizing refractive index modulation in a non-volatile manner.

Such materials could exist in stable amorphous and crystalline states with a stark difference in refractive index between the two phases [165]. Chalcogenides have high dielectric indices supporting Mie-type resonances on the subwavelength scale and have been used extensively for realizing tunable and reconfigurable metasurfaces [166–169].

However, we should note that achieving a nonreciprocal metasurface based on the spatiotemporal modulation requires the permittivity to change temporally and spatially. Ideally, the time modulation of adjacent unit cells should be designed to have a position-dependent phase difference of *ϕ*(Δ*x*) to create a permittivity distribution with $\varepsilon \left(x\right)=\varepsilon_{1}+\varepsilon_{m}\cos\left(\omega_{m}t+\phi \left(x\right)\right)$ , which is difficult to realize in practice. Because of the difficulties associated with the implementation of time modulations and the limited modulation frequencies that are practically available, spatiotemporal metasurfaces has been mostly limited to theoretical investigations and realizing these structures is still open for investigation.

### 2.5 Unidirectional edge states in topological photonic structures

Topological photonic structures are borrowed from topological insulators in condensed matter physics due to the similarity of the electronic band diagrams to dispersion diagrams in photonic crystals. Similar to the topological insulators, where the nontrivial topology of band diagrams for an infinite crystal is identified with unidirectional edge states on the boundary of a finite crystal, photonic topological insulators (PTI) are also recognized with the presence of unidirectional waves that propagate along the edge of the photonic crystals [170]. In general, PTI can be divided into two categories of time-reversal symmetric and time-reversal broken systems. Time-reversal broken PTI have non-zero Chern number – a topological invariant defined based on how the state of the structure changes over a close loop in the Brillouin zone [171, 172]. Such PTI were demonstrated with photonic crystals comprising magneto-optic or gyromagnetic properties in the presence of the magnetic field bias [173–175]. PTI with non-zero Chern numbers were also demonstrated by dynamic modulation of a resonator lattice where the time evolution of the lattice resembles that of the electron sites in a simplified model of solids [176]. Furthermore, due to the analogy between equations governing the time evolution of quantum states with the equations describing the propagation of waves, lattices of waveguides with broken z-symmetry are also utilized to mimic time-symmetry broken systems and realize PTI with non-zero Chern number [177]. Such TPI that operates based on breaking time-reversal symmetry are nonreciprocal systems.

In PTI that are time-reversal symmetric, additional degrees of freedom are introduced into the photonic crystals, such as polarization or angular momentum, that mimic the effect of the spin or other degrees of freedom in topological insulators, and they mimic the time-reversal symmetric topological insulators and quantum spin hall effect [178, 179]. In such PTI, pairs of unidirectional edge states propagating in opposite directions appear that are differentiated with opposite polarization or angular momentum. Such PTI were demonstrated first in two-dimensional arrays resonators that are coupled to each other with optical waveguides, where the propagation direction in each ring resembles the effect of spin [180, 181]. Time-reversal symmetric PTI are also demonstrated in photonic crystals with honeycomb lattices. In these structures, lattice sites and lattice constants in the photonic crystal are designed such that it creates nontrivial band gaps.3A trivial band gaps occur when two modes with the same properties and the opposite momentum destructively interfere while in a nontrivial bandgap, the interference happen between modes that have other differences aside from opposite momentum. Unidirectional edge states then appear along the edges between a PTIwith a nontrivial band gap and simple photonic crystal with a trivial bandgap. Honeycomb crystal structures of metacrystals at microwave frequencies are used to create nontrivial topology based on the asymmetry between two modes of the metacrystal waveguide [182, 183], and it leads to unidirectional edge states with a particular mode of the photonic crystal waveguide. Furthermore, dielectric photonic crystals and membrane photonic crystals are also reported for creating PTI where the angular moment in each unit cell plays the role of the additional degree of freedom and creates the nontrivial band gaps and unidirectional edge states for distinct angular momentum of EM fields [184–186].

We should note that despite time-reversal broken PTI that are nonreciprocal systems, time-reversal symmetric PTI are reciprocal, and the unidirectional edge states exist for a certain polarization or modes with certain angular momentum. It has been discussed in the literature that some unidirectional edge states in the time-reversal symmetric PTI are not immune from scattering when the discontinuity at the edge can scatter to other polarizations or angular momentum [176].

Unidirectional edge states attracted a lot of attention over the last decade since they provide propagation of EM waves that are immune from back scattering, and presence of defects does not lead to the scattering of these states. Such states also are used for designing lasers whose mode is not affected with defects [187]. Unidirectional edge states only exist on the boundary of artificial structures, which can be realized using photonic crystals with specific geometries. Therefore, they are promising candidates when unidirectional propagation of EM waves in waveguide systems is required. A detailed description of topological edge states and how they can be realized can be found in other review papers [171, 172, 188].

## 3 AT in reciprocal structures

Nonreciprocal transmission requires an external system to create a static or dynamic bias field. Therefore, realizing nonreciprocal metasurfaces in micro/nanoscale such that they can be integrated with other optical components and form a compact optical device remains a challenge. However, power transmission by means of EM waves between two sources of energy can be asymmetric for a reciprocal structure. If one models the system with a network where each mode of the transmitter/receiver is considered as a port, then reciprocity implies a symmetric transmission between two ports. However, if the transmitter or receiver host multiple modes, and we consider the transmission as the total power received from all ports of the receiver side, then similar excitation of the transmitter and receiver side, can lead to different transmission. For waves propagating in the free space, EM modes with a fixed frequency are distinguished based on their direction of propagation and their polarization. Therefore, metasurfaces that can couple the energy of the incident wave to multiple EM modes and transmit power through those modes can provide AT for the total transmission. In such metasurfaces varying the mode of excitation obviously change the total transmission, and for AT in reciprocal structures in a metasurface we consider a same excitation from both sides of the metasurface results in different transmission. Therefore, AT in a reciprocal structure is restricted to a specific type of incident wave, such as a fixed incident angle or polarization. For AT in a reciprocal metasurface, input and output EM waves must be able to exist in different states (modes) such that metasurface transform state of the incident wave to a secondary state (e.g. different polarization or different propagation direction). Besides, there must be an asymmetry in the geometry such that interactions between modes and excitation of modes results in a high transmission only for one direction and not the other. Metasurfaces with reciprocal AT can be divided into two types. Either they are designed based on polarization conversion, or they are based on diffraction orders in the metasurface and transmitting light through higher diffraction orders. This section reviews both types of structures and provides general rules for designing such asymmetric metasurfaces.

### 3.1 AT in polarizer metasurfaces

A metasurface that can change the polarization of light and lacks mirror symmetry in its geometry can provide AT for specific polarization of light. To illustrate the reason for AT in such structures, we consider the transmission matrix of two orthogonal polarizations as

(9) $$\left[\begin{array}{c} {E_{1}}_{p_{1}}\\ {E_{1}}_{p_{2}} \end{array}\right]=\left[\begin{array}{cc} t_{p_{1}p_{1}}^{f} & t_{p_{1}p_{2}}^{f}\\ t_{p_{2}p_{1}}^{f} & t_{p_{2}p_{2}}^{f} \end{array}\right]\left[\begin{array}{c} {E_{2}}_{p_{1}}\\ {E_{2}}_{p_{2}} \end{array}\right]$$

Where ${E_{1}}_{p_{1}\left(p_{2}\right)}$ and ${E_{2}}_{p_{1}\left(p_{2}\right)}$ are complex numbers representing complex amplitude of electric fields corresponding to *p*_1_(*p*_2_) polarizations on two sides of the metasurface. The transmission matrix in the backward direction can be calculated based on the inverse of the transmission matrix in the forward direction. In a reciprocal structure, transmission matrix for backward transmission is not independent from forward transmission matrix and it can be written as

(10) $$T^{b}=\left[\begin{array}{cc} t_{p_{1}p_{1}}^{b} & t_{p_{1}p_{2}}^{b}\\ t_{p_{2}p_{1}}^{b} & t_{p_{2}p_{2}}^{b} \end{array}\right]=\left[\begin{array}{cc} t_{p_{1}p_{1}}^{f} & -t_{p_{2}p_{1}}^{f}\\ -t_{p_{1}p_{2}}^{f} & t_{p_{2}p_{2}}^{f} \end{array}\right].$$

We note that the transmission matrix for backward excitation can be fully described by knowing the forward transmission matrix. Reciprocity implies that the diagonal elements of the forward and backward transmission matrices are equal, i.e., $t_{p_{1}p_{1}}^{f}=t_{p_{1}p_{1}}^{b}$ and $t_{p_{2}p_{2}}^{f}=t_{p_{2}p_{2}}^{b}$ . However, total transmission also depends on off-diagonal components in the transmission matrix which are not necessarily equal. One can show that in a reciprocal metasurface, the difference between total forward and backward transmission when excited with a fixed polarization from both sides is $|\Delta T|={\left|t_{p_{1}p_{2}}\right|}^{2}-{\left|t_{p_{2}p_{1}}\right|}^{2}$ .

Therefore, to have AT for a specific type of polarization, the metasurface must change the polarization of the incident wave, and polarization conversion from *p*_1_ → *p*_2_ and *p*_2_ → *p*_1_ must be different.

**Figure 9:**
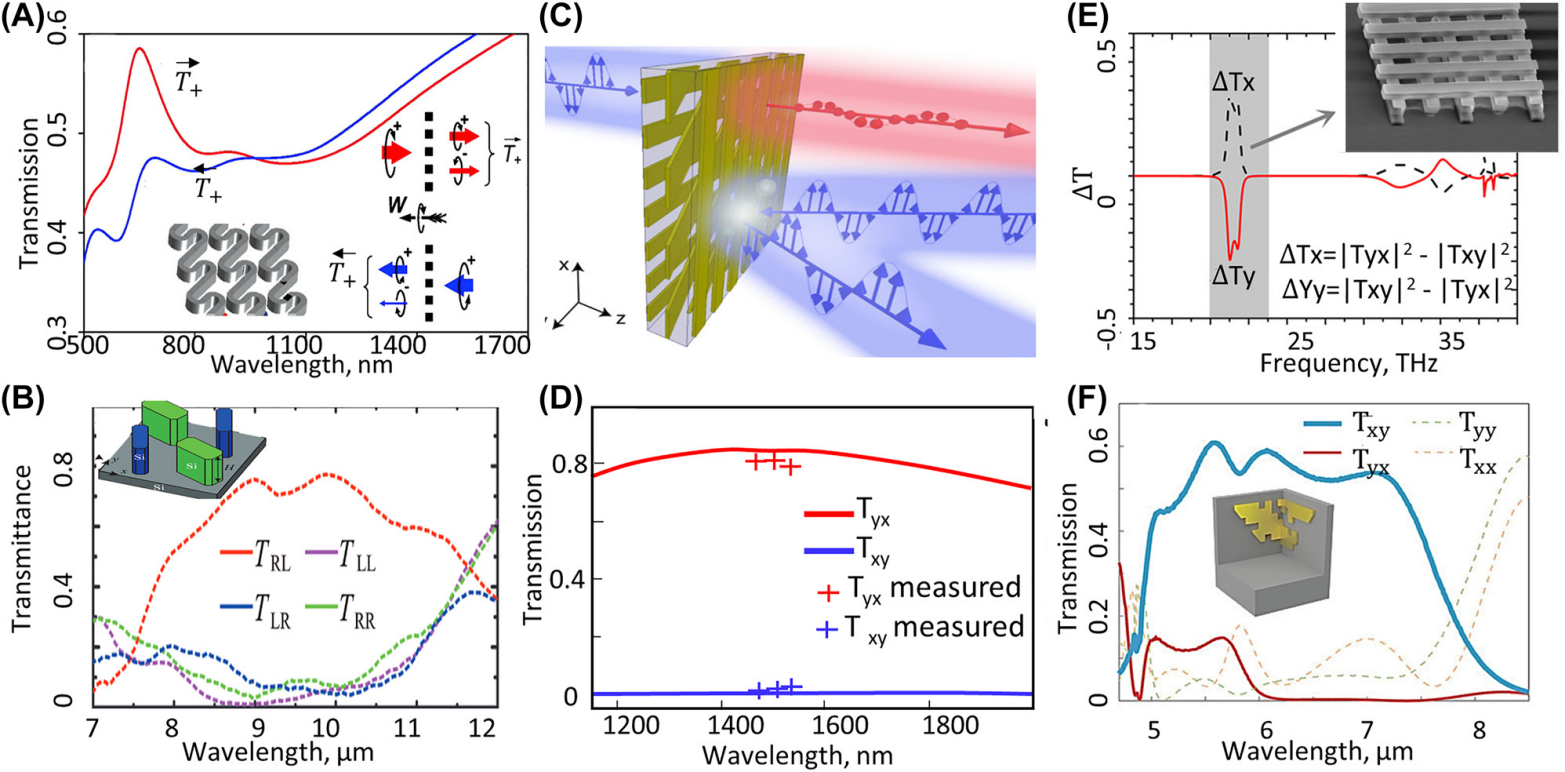
AT based on polarization conversion metasurfaces. (A) shows AT of circularly polarized light for excitation from opposite directions identified with blue and red, and the inset shows the schematics of the plasmonic chiral metasurface (adopted with permission from [74]). Here subscripts + and − demonstrate opposite direction of the circular polarization and the superscript arrow demonstrate the direction of propagation. (B) shows the matasurface designed based on a single layer metasurface containing silicon pillars with asymmetric distribution that creates AT for circularly polarized light and the measurement data for transmission of circularly polarized light where R and L represent right handed and left handed circular polarizations (adopted with permission from [76]). (C) and (D) shows the schematics of the metasurface for wideband AT of linearly polarized light in IR wavelengths based on three layers of gold nanogratings separated with silica layers, and the simulated and measured transmission for excitation with different linear polarizations (adopted with permission from [195]). (E) Three-layer metasurface for AT of linearly polarized light at Terahertz fabricated with direct laser writing [197]. (F) Wide band AT for linearly polarized light based on optimized unit cell that are fabricated with projection lithography method [198]. (E and F are adopted from [197] and [198]).

AT based on polarization conversion has been demonstrated for circularly polarized [73, 74, 189], [190], [191] and linearly polarized light [71, 72, 192], [193], [194], [195]. AT based on polarization conversion was first demonstrated in a single layer twisted plasmonic metasurface for circular polarization (Figure 9A) [75, 196]. It was shown that the lack of rotational symmetry in this metasurface results in polarization transformation, and since the mirror image of the unit cell was not congruent with itself, conversion efficiency from RCP to LCP and LCP to RCP were different from each other resulting in 25% asymmetry in the total transmission. To enhance the contrast between forward and backward transmission for a fixed polarization Zhao et al. utilized a stack of nanorods on top of each other. It was numerically shown that as the number of layers in the stack increases, asymmetry in the transmission increases and the bandwidth of the AT also increases [73]. Zhang et al. designed an all-dielectric metasurface that provide wavefront shaping and asymmetric transmission of circularly polarized light [76]. They designed supercells containing four silicon pillars arranged in an asymmetric manner that results in asymmetric polarization conversion and therefore asymmetric transmission, Figure 9B. Furthermore, the authors implemented the Pancharatnam–Berry design principles [199] where orientation of ellipsoid pillars with respect to the polarization angle is used to introduce the required phase difference among different unit cells resulting in simultaneous beam and wavefront shaping. A similar principle has been applied to achieving AT for a linearly polarized light. AT for linearly polarized light requires the transmission coefficient of *x*-polarization to *y*-polarization, $\left|T_{xy}\right|$ , to be different from $\left|T_{yx}\right|$ . Such asymmetry in the transmission matrix for normal incident wave cannot be realized in single-layer metasurfaces, and two or more layers are required to realize this condition. Menzel et al. demonstrated AT for linearly polarized light using a metallic double-layer metasurface comprising nanowires on one layer and an L-shaped metallic structure on the second layer [193], resulting in 25% percent difference between forward and backward total transmission. To enhance the asymmetry in the transmission and decrease the sensitivity to the incident angle, Zhang et al. implemented three layers of gold gratings stacked on top of each other, separated by a silica spacer, Figure 9C and D, and they demonstrated more than 80 percent asymmetry in the transmission for a wide range of incident angles [195]. Similarly, stack of three gratings with different orientation was also used for creating AT of linearly polarized light for THz frequencies [197], Figure 9E. Generalizing layered structures to 3D unit cells with arbitrary shapes could further improve the AT for a fixed linear polarization. For example, 3D unit cells, Figure 9F, that are designed based on optimization methods and fabricated with membrane projected lithography, have been used to realize AT with wide bandwidth [198]. We should note that polarization converting metasurfaces and metamaterials are not necessary for creating AT based on the polarization conversion and combination of birefringent materials with polarizers can also create this behavior [200].

### 3.2 AT based on higher diffraction orders

Scattering of EM waves into several diffraction orders is a well-known behavior in periodic structures leading to the ubiquitous usage of diffraction gratings in various applications. In conventional gratings, one has limited control over how energy is distributed among various diffraction orders. However, by engineering the arrangement of periodic patterns on the subwavelength scale, one can design metagratings for controlling the energy distribution among various diffraction orders. In metagrating, diffraction orders arise from supercells containing subwavelength unit cells, and one can manipulate the geometry of unit cells and super cells to suppress or enhance the power coupled to the desired diffraction orders. In a metagrating lacking mirror symmetry, the energy distribution over multiple diffraction orders could be different for excitation from opposite directions, which can be used for realizing AT. This can be best understood in terms of the generalized scattering matrix which represents the scattering parameters of all diffraction orders. Consider the generalized scattering matrix to be represented by:

(11) $$S=\left[\begin{array}{cc} R^{\text{f}} & T^{\text{b}}\\ T^{\text{f}} & R^{\text{b}} \end{array}\right]$$

where $R^{\text{f}\left(b\right)}$ and $T^{\text{f}\left(b\right)}$ are transmission and reflection block matrices for two opposite sides of the metasurface, front side (f) and back side (b). The *mn* ^*th*^ element $R_{mn}^{\text{f}\left(b\right)}\left(T_{mn}^{\text{f}\left(b\right)}\right)$ designates the reflection (or transmission) to diffraction order *m* when incident wave is a plane wave corresponding to diffraction order *n*. In reciprocal strucures, the scattering matrix is symmetric $T_{mn}^{b}=T_{nm}^{f}$ , however, the total transmission is a summation over all the transmitted propagating diffraction orders. For example, when the incident wave is a plane wave in the direction of *I*th the total forward and backward transmission are:

(12) $$T_{I}^{\text{F}}=\underset{j}{\sum }{\left|T_{jI}^{\text{f}}\right|}^{2},\;T_{I}^{\text{B}}=\underset{j}{\sum }{\left|T_{jI}^{\text{b}}\right|}^{2}$$

Therefore, one can achieve AT by designing the metagrating in a way that $\left|T_{jI}^{b}\right|$ has small values for all diffraction orders (*j*) while ${\left|T_{jI}^{\text{f}}\right|}^{2}$ is as large as possible. One can see that because of reciprocity $T_{II}^{\text{f}}=T_{II}^{\text{b}}$ , which means suppression of the zeroth order diffraction order is a necessary condition for AT. Besides, metagratings that are symmetric along the direction of transmission cannot create AT since in such structures $T_{j_{I}}^{\text{f}}=T_{j_{I}}^{\text{b}}$ .

Satisfying these conditions requires designing asymmetric structures that can couple energy to higher diffraction orders and show high transmission from one direction, while they reflect all the energy for illumination from the opposite direction. AT based on diffraction orders was demonstrated by Serebryannikov et al., for thick photonic crystal slabs with asymmetric corrugations on two sides of a slab [78], [79], [80, 83, 201], where it was shown that knowing the equifrequency surfaces of a photonic crystal, one can add corrugations with different periodicities on two sides of the photonic crystal slab such that incident light could couple to the propagating modes of the photonic crystal slab from one side and scatter to higher order diffraction orders from the other side. For thick photonic crystal slabs one can design the gratings on two sides of the thick slab independent from each other, since the gratings do not affect the photonic crystal propagating modes, and one can design these structures based on the equifrequency surfaces of a photonic crystal and required condition for coupling a free space wave to these bulk modes.

Later, optically thin structures such as double-layer gratings and asymmetric gratings were suggested for realizing asymmetric transmission for optical and microwave frequencies [81, 82, 84, 86], [87], [88], [89], [90], [91, 202], [203], [204], [205]. The main design principle for realizing AT with thin asymmetric gratings, is suppressing transmission through the zeroth diffraction order and utilizing leaky waves that can propagate along the surface and leak energy through higher diffraction orders. Furthermore, because of the asymmetric geometry of such structures, leaky modes in these thin structures can only be excited from one direction which means high transmission happens only for excitation from one direction.

**Figure 10:**
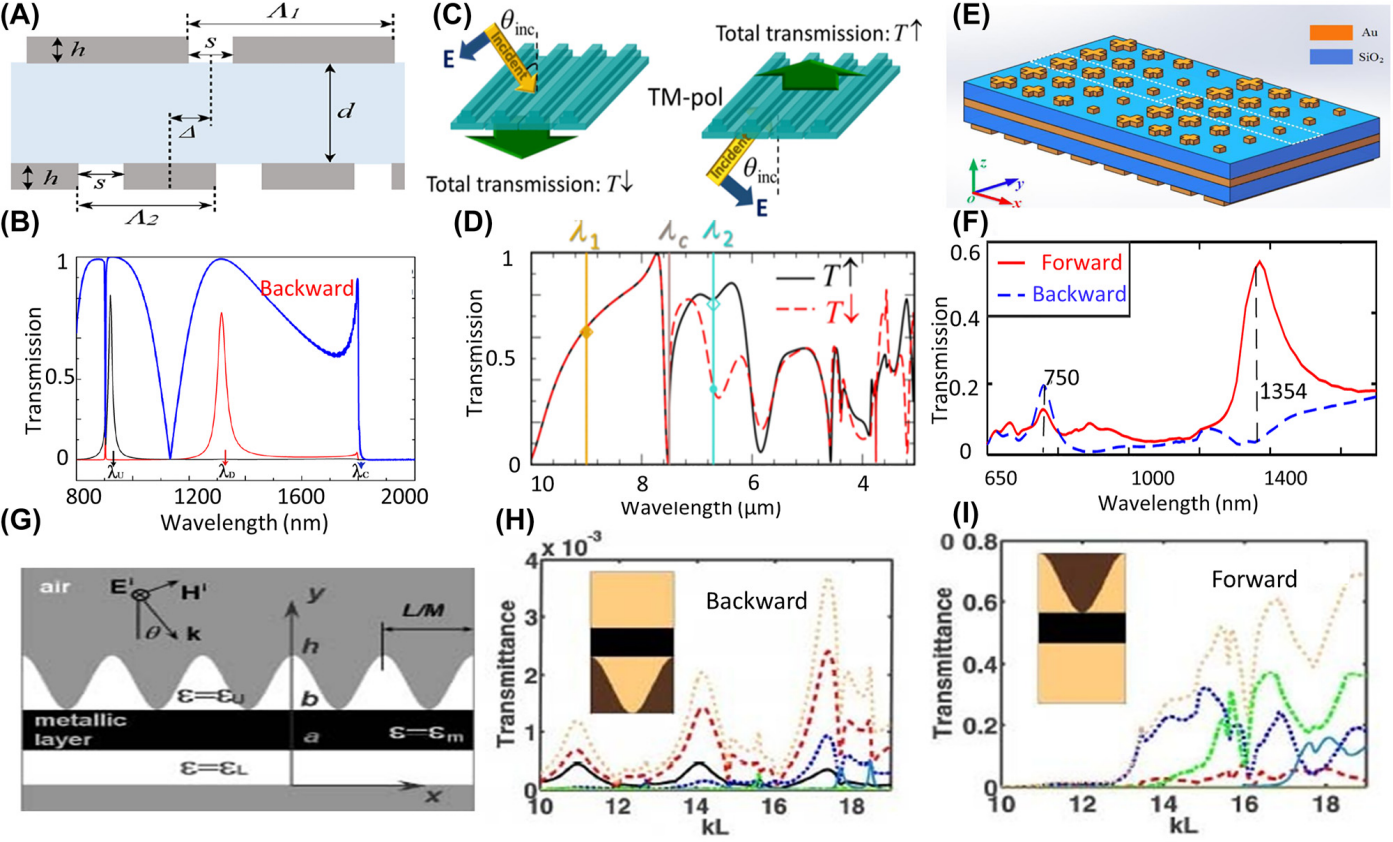
AT based on diffraction orders. (A) and (B) Using asymmetric silver gratings with different periodicities on two side of a thin silica layer and the corresponding forward and backward transmission [82]. (C) schematics and (D) results of an all-dielectric metagrating that creates AT based on the higher diffraction order (adopted with permission from [204]). (E) and (F) shows the metasurface comprising combination of a gradient metasurface and gold grating to create AT utilizing asymmetric excitation of surface plasmon resonances, and the corresponding transmission response at visible and IR [91]. (G) shows a sinusoidal dielectric grating and thin metallic on top of a thin metallic film used for AT based on higher order diffraction orders, and (H) and (I) shows the transmission for multiple diffraction orders. Dotted yellow line represent total transmission and other colors represent transmission through different diffraction orders [201].

For example, Figure 10A and B shows the double-layer structure in Ref. [82] where the grating from one side creates higher diffraction orders that can couple to leaky surface plasmons in the second grating resulting in high transmission. However, excitation from the other direction will be reflected since it cannot couple to the leaky waves. Furthermore, AT from an all-dielectric metagrating based on the higher diffraction order is demonstrated, Figure 10C and D, in [204] and the importance of the excitation of propagating diffraction order is explained based on the scattering matrix of all the existing modes, and it is demonstrated that plasmonic modes are not necessary for AT in such structures. Similarly, in [92] a combination of gradient metasurfaces and nanogratings, Figure 10E, are used to couple the normal incident light to the surface plasmon waves that can later leak out through the second grating through higher diffraction orders while the normal incident light from the opposite direction cannot excite the surface plasmon waves resulting in negligible transmission as shown in Figure 10F. In [201], it is theoretically shown that a periodic structure on top of a metallic slab, Figure 10G, results in AT of higher order diffraction because of asymmetric excitation of surface plasmons in such structures, Figure 10H and I. Except for a few 2D-metasurfaces reported for AT [88–90], most of the literature showing AT based on diffraction orders in metasurfaces has been limited to 1-D gratings, and they provide asymmetric transmission for only TE (transverse electric) or TM (transverse magnetic) polarization. The role of higher diffraction orders in most of the demonstrations is not clarified which could lead to confusion and results that cannot be realized and measured. Several metasurfaces proposed for AT in asymmetric structures are proposed based on a substrate, and they showed AT results in simulations. However, one should note that in various scenarios, the diffraction orders are propagating only inside the substrate because of its higher refractive index and such higher diffraction orders can never transmit from substrate to the air at the back side of the substrate. In asymmetric metagratings, the transmitted light for each wavelength will propagate in a different direction as it is forced from the physics of diffraction in periodic structures. Therefore, it is important to identify the direction of propagation of the output beam and one should consider this fact in scenarios reporting wideband AT. 

## 4 Applications and outlook for reciprocal and nonreciprocal AT

The main application for nonreciprocal AT is creating isolators and circulators. Although bulky isolators based on Faraday rotation are already commercialized, creating thin surfaces that operate as isolators based on Faraday rotation is quite challenging, as discussed in Section 2.2. Besides, one of the main motivations for developing metasurfaces is creating compact devices for real-life applications generally on the micro and nanoscale. However, AT in metasurfaces based on magneto-optic effect relies on large external magnetic field biases, which means they cannot be used in a compact optical device unless magnetic field bias could also be realized on a micro/nano scale. Engineering materials with large Verdet constants could lead to more efficient usage of these nonreciprocal structures in nanophotonics. Nonreciprocal metasurfaces based on nonlinear materials are intensity-dependent and could only isolate high-intensity lasers. Such metasurfaces have been studied theoretically, and the feasibility of fabrication and measurement of such metasurfaces is still open for discussion. Another limitation in nonreciprocal metasurfaces operating based on the magneto-optic effect and nonlinear properties are that they require resonances with very high Q-factors to enhance the interaction between EM waves and the material. Being dependent on high-Q resonances limits their performance to a narrow band of frequencies and incident angles.

Utilizing spatiotemporal modulation for achieving a nonreciprocal metasurface has attracted a lot of attention over the last decade as a magnetic-free approach for generating nonreciprocal metasurfaces. However, establishing the required spatiotemporal modulation in the permittivity is challenging as discussed in Section 2.4. New techniques for providing fast time-modulation at optical behavior could lead to more broad usage of these structures when nonreciprocal behavior is required.

Furthermore, creating such modulations usually requires adding more layers to the structure due to which the nonreciprocal behavior of such metasurfaces is usually demonstrated in the reflection mode where despite showing a nonreciprocal behavior, they do not provide AT. The other limitation in these structures is that because of time modulation, the input and output waves have different frequencies and direction of propagation, (see Figure 8E and F) therefore they can be used in applications where frequency of the input light is different with output light.

On the other hand, reciprocal AT does not require any external system for bias and modulation, nor does it depend on the intensity of the input power. However, in contrast to nonreciprocal metasurfaces, reciprocal AT cannot create complete isolation, meaning that if one puts an infinite mirror in front of such a structure, eventually, all the light will be reflected. Nonetheless, such structures could be useful for decoupling two different paths of light or different sources from each other, and they can still isolate some part of the undesired light that could excite the metasurface from a third direction. Metasurfaces with AT based on polarization are of interest for polarization conversion and circular dichroism applications, and they can be used in polarization multiplexing for holography application since they encode different information on polarization from opposite directions.

Metasurfaces and metagratings that provide AT of EM waves through control of higher diffraction orders are attractive candidates to be used in communication applications by providing beam multiplexing, splitting and convergence. For example, asymmetric metagratings can be utilized in applications where wavelength multiplexing is required, and they can be used in the transmission mode for the light from one side and in the reflection mode for the light coming from the opposite side without mixing the lights that are coming from opposite directions, which could lead to more compact devices. Furthermore, since metagratings with AT could act as a mirror from one side, they can be used in designing laser integrated metagratings and provide more control on the output beam of lasing systems. Asymmetric metagratings can also be utilized for cloaking and shielding at small scales since they transmit the EM waves only from one direction. Redirecting the propagation direction of light could also benefit photovoltaic devices by enhancing the absorption. New generations of flexible, ultrathin solar cells containing subwavelength absorbing layers naturally exhibit low efficiencies. Metagratings with AT responses that are discussed here could be integrated with various photovoltaic material platforms to enhance absorption by redirecting light to higher diffraction orders and steep angles by trapping a higher proportion of the incoming light.

In general, most of the nonreciprocal systems discussed in this review for AT using metasurfaces are sensitive to the incident angles since either they rely on high-*Q* resonances or surface waves to create spatiotemporal modulation and therefore are sensitive to the incident angles. On the other hand, reciprocal structures that create AT based on transmitting light to other diffraction orders can theoretically create AT for a wider range of incident angles, but they are still limited to incident angles due to the Brag condition. Besides, in these structures, the incident angle of the backward excitation is the same as the incident angle of the forward excitation, and this angle is different from the angle of transmitted light. The limitation to the incident angles for AT in both reciprocal and nonreciprocal structures cause challenges for applications where isolation for a large range of incident angles is required, such as trapping light for solar cell applications or creating EM shielding. Realizing a large, pixelated area containing distinct metasurfaces designed for different angles at each pixel could slightly improve this sensitivity.

Therefore, it is clear that nanophotonic devices and components exhibiting asymmetric transmission of light will be of immense societal importance and impact going forward in a myriad of industries, from telecommunication and computing to photovoltaics and laser sciences. While the prevailing technology platforms in each industry may not be clear for long-term future development, the role that asymmetric transmission devices and concepts will play within each platform to enhance relevant performance metrics for each specific application type is profound.

## References

[1] Chen H. T., Taylor A. J., Yu N. (2016). A review of metasurfaces: physics and applications. *Rep. Prog. Phys.*.

[2] Chen W. T., Zhu A. Y., Capasso F. (2020). Flat optics with dispersion-engineered metasurfaces. *Nat. Rev. Mater.*.

[3] Kildishev A. v., Boltasseva A., Shalaev V. M. (2013). Planar photonics with metasurfaces. *Science*.

[4] Juliano Martins R., Marinov E., Youssef M. A. B. (2022). Metasurface-enhanced light detection and ranging technology. *Nat. Commun*..

[5] Neshev D., Aharonovich I. (2018). Optical metasurfaces: new generation building blocks for multi-functional optics. *Light. Sci. Appl.*.

[6] Huang L., Zhang S., Zentgraf T. (2018). Metasurface holography: from fundamentals to applications. *Nanophotonics*.

[7] Yulaev A., Zhu W., Zhang C. (2019). Metasurface-integrated photonic platform for versatile free-space beam projection with polarization control. *ACS Photonics*.

[8] Ding Y., Chen X., Duan Y. (2022). Metasurface-dressed two-dimensional on-chip waveguide for free-space light field manipulation. *ACS Photonics*.

[9] Martins T., Cui Y., Gholipour B., Ou J. Y., Frazão O., MacDonald K. F. (2021). Fiber-integrated phase change metasurfaces with switchable group delay dispersion. *Adv. Opt. Mater.*.

[10] Bi L., Hu J., Jiang P. (2011). On-chip optical isolation in monolithically integrated non-reciprocal optical resonators. *Nat. Photonics*.

[11] Dötsch H., Bahlmann N., Zhuromskyy O. (2005). Applications of magneto-optical waveguides in integrated optics: Review. *JOSA B*.

[12] Kutsaev S. v., Krasnok A., Romanenko S. N., Smirnov A. Yu., Taletski K., Yakovlev V. P. (2021). Up‐and‐coming advances in optical and microwave nonreciprocity: from classical to quantum realm. *Adv. Photonics Res.*.

[13] Jiao Y. F., Zhang S. D., Zhang Y. L., Miranowicz A., Kuang L. M., Jing H. (2020). Nonreciprocal optomechanical entanglement against backscattering losses. *Phys. Rev. Lett.*.

[14] Caloz C., Alù A., Tretyakov S., Sounas D., Achouri K., Deck-Léger Z. L. (2018). Electromagnetic nonreciprocity. *Phys. Rev. Appl.*.

[15] Asadchy V. S., Mirmoosa M. S., Diaz-Rubio A., Fan S., Tretyakov S. A. (2020). Tutorial on electromagnetic nonreciprocity and its origins. *Proc. IEEE*.

[16] Adam J. D., Davis L. E., Dionne G. F., Schloemann E. F., Stitzer S. N. (2002). Ferrite devices and materials. *IEEE Trans. Microw. Theor. Tech.*.

[17] Shoji Y., Mizumoto T., Yokoi H., Hsieh I. W., Osgood R. M. (2008). Magneto-optical isolator with silicon waveguides fabricated by direct bonding. *Appl. Phys. Lett.*.

[18] Sun X. Y., Du Q., Goto T. (2015). Single-step deposition of cerium-substituted yttrium iron garnet for monolithic on-chip optical isolation. *ACS Photonics*.

[19] Du Q., Wang C., Zhang Y. (2018). Monolithic on-chip magneto-optical isolator with 3 dB insertion loss and 40 dB isolation ratio. *ACS Photonics*.

[20] Zhang C., Dulal P., Stadler B. J. H., Hutchings D. C. (2017). Monolithically-integrated TE-mode 1D silicon-on-insulator isolators using seedlayer-free garnet. *Sci. Rep.*.

[21] Sung S. Y., Qi X., Stadler B. J. H. (2005). Integrating yttrium iron garnet onto nongarnet substrates with faster deposition rates and high reliability. *Appl. Phys. Lett*..

[22] Lodewijks K., Maccaferri N., Pakizeh T. (2014). Magnetoplasmonic design rules for active magneto-optics. *Nano Lett*..

[23] Belotelov V. I., Akimov I. A., Pohl M. (2011). Enhanced magneto-optical effects in magnetoplasmonic crystals. *Nat. Nanotechnol*..

[24] González-Díaz J. B., Sepúlveda B., García-Martín A., Armelles G. (2010). Cobalt dependence of the magneto-optical response in magnetoplasmonic nanodisks. *Appl. Phys. Lett.*.

[25] Chin J. Y., Steinle T., Wehlus T. (2013). Nonreciprocal plasmonics enables giant enhancement of thin-film Faraday rotation. *Nat. Commun.*.

[26] Kreilkamp L. E., Belotelov V. I., Chin J. Y. (2014). Waveguide-plasmon polaritons enhance transverse magneto-optical kerr effect. *Phys. Rev. X*.

[27] Chen S., Fan F., Wang X., Wu P., Zhang H., Chang S. (2015). Terahertz isolator based on nonreciprocal magneto-metasurface. *Opt. Express*.

[28] Khokhlov N. E., Prokopov A. R., Shaposhnikov A. N. (2015). Photonic crystals with plasmonic patterns: novel type of the heterostructures for enhanced magneto-optical activity. *J. Phys. D Appl. Phys.*.

[29] Floess D., Hentschel M., Weiss T. (2017). Plasmonic analog of electromagnetically induced absorption leads to giant thin film Faraday rotation of 14°. *Phys. Rev. X*.

[30] Sepúlveda B., González-Díaz J. B., García-Martín A., Lechuga L. M., Armelles G. (2010). Plasmon-induced magneto-optical activity in nanosized gold disks. *Phys. Rev. Lett*..

[31] Barsukova M. G., Musorin A. I., Shorokhov A. S., Fedyanin A. A. (2019). Enhanced magneto-optical effects in hybrid Ni-Si metasurfaces. *APL Photonics*.

[32] Barsukova M. G., Shorokhov A. S., Musorin A. I., Neshev D. N., Kivshar Y. S., Fedyanin A. A. (2017). Magneto-optical response enhanced by Mie resonances in nanoantennas. *ACS Photonics*.

[33] Christofi A., Kawaguchi Y., Alù A., Khanikaev A. B. (2018). Giant enhancement of Faraday rotation due to electromagnetically induced transparency in all-dielectric magneto-optical metasurfaces. *Opt. Lett*..

[34] Abendroth J. M., Solomon M. L., Barton D. R., el Hadri M. S., Fullerton E. E., Dionne J. A. (2020). Helicity-preserving metasurfaces for magneto-optical enhancement in ferromagnetic [Pt/Co]N films. *Adv. Opt. Mater.*.

[35] Mahmoud A. M., Davoyan A. R., Engheta N. (2015). All-passive nonreciprocal metastructure. *Nat. Commun*..

[36] Fan L., Wang J., Varghese L. T. (2012). An all-silicon passive optical diode. *Science*.

[37] Yang K. Y., Skarda J., Cotrufo M. (2020). Inverse-designed non-reciprocal pulse router for chip-based LiDAR. *Nat. Photonics*.

[38] Yu Y., Chen Y., Hu H., Xue W., Yvind K., Mork J. (2015). Nonreciprocal transmission in a nonlinear photonic-crystal Fano structure with broken symmetry. *Laser. Photon. Rev.*.

[39] Xu M., Wu J., Wang T., Hu X., Jiang X., Su Y. (2013). Push-pull optical nonreciprocal transmission in cascaded silicon microring resonators. *IEEE Photonics J.*.

[40] Sounas D. L., Soric J., Alù A. (2018). Broadband passive isolators based on coupled nonlinear resonances. *Nat. Electron.*.

[41] Lawrence M., Barton D. R., Dionne J. A. (2018). Nonreciprocal flat optics with silicon metasurfaces. *Nano Lett*..

[42] Mekawy A., Sounas D. L., Alù A. (2021). Free‐space nonreciprocal transmission based on nonlinear coupled fano metasurfaces. *Photonics*.

[43] Jin B., Argyropoulos C. (2020). Self-induced passive nonreciprocal transmission by nonlinear bifacial dielectric metasurfaces. *Phys. Rev. Appl.*.

[44] Wan C., Horak E. H., King J. (2018). Limiting optical diodes enabled by the phase transition of vanadium dioxide. *ACS Photonics*.

[45] Hajian H., Ghobadi A., Serebryannikov A. E., Butun B., Vandenbosch G. A. E., Ozbay E. (2019). VO 2 -hBN-graphene-based bi-functional metamaterial for mid-infrared bi-tunable asymmetric transmission and nearly perfect resonant absorption. *J. Opt. Soc. Am. B*.

[46] Karvounis A., Ou J.-Y., Wu W., MacDonald K. F., Zheludev N. I. (2015). Nano-optomechanical nonlinear dielectric metamaterials. *Appl. Phys. Lett.*.

[47] Zhang J., MacDonald K. F., Zheludev N. I. (2013). Nonlinear dielectric optomechanical metamaterials. *Light. Sci. Appl.*.

[48] Ruesink F., Miri M. A., Alù A., Verhagen E. (2016). Nonreciprocity and magnetic-free isolation based on optomechanical interactions. *Nat. Commun.*.

[49] Manipatruni S., Robinson J. T., Lipson M. (2009). Optical nonreciprocity in optomechanical structures. *Phys. Rev. Lett.*.

[50] Shi Y., Yu Z., Fan S. (2015). Limitations of nonlinear optical isolators due to dynamic reciprocity. *Nat. Photonics*.

[51] Lira H., Yu Z., Fan S., Lipson M. (2012). Electrically driven nonreciprocity induced by interband photonic transition on a silicon chip. *Phys. Rev. Lett*..

[52] Kang M. S., Butsch A., Russell P. S. J. (2011). Reconfigurable light-driven opto-acoustic isolators in photonic crystal fibre. *Nat. Photonics*.

[53] Pandey A., Dwivedi S., Zhenzhou T., Pan S., van Thourhout D. (2021). Nonreciprocal light propagation in a cascaded all-silicon microring modulator. *ACS Photonics*.

[54] Lin Q., Wang J., Fan S. (2019). Compact dynamic optical isolator based on tandem phase modulators. *Opt. Lett*..

[55] Sounas D. L., Caloz C., Alù A. (2013). Giant non-reciprocity at the subwavelength scale using angular momentum-biased metamaterials. *Nat. Commun.*.

[56] Sounas D. L., Alù A. (2014). Angular-momentum-Biased nanorings to realize magnetic-free integrated optical isolation. *ACS Photonics*.

[57] Tian H., Liu J., Siddharth A. (2021). Magnetic-free silicon nitride integrated optical isolator. *Nat. Photonics*.

[58] Yu Z., Fan S. (2009). Complete optical isolation created by indirect interband photonic transitions. *Nat. Photonics*.

[59] Shi Y., Han S., Fan S. (2017). Optical circulation and isolation based on indirect photonic transitions of guided resonance modes. *ACS Photonics*.

[60] Shi Y., Fan S. (2016). Dynamic non-reciprocal meta-surfaces with arbitrary phase reconfigurability based on photonic transition in meta-atoms. *Appl. Phys. Lett.*.

[61] Shaltout A., Kildishev A., Shalaev V. (2015). Time-varying metasurfaces and Lorentz non-reciprocity. *Opt. Mater. Express*.

[62] Guo X., Ding Y., Duan Y., Ni X. (2019). Nonreciprocal metasurface with space–time phase modulation. *Light. Sci. Appl.*.

[63] Hadad Y., Sounas D. L., Alu A. (2015). Space-time gradient metasurfaces. *Phys. Rev. B: Condens. Matter Mater. Phys.*.

[64] Sounas D. L., Alù A. (2017). Non-reciprocal photonics based on time modulation. *Nat. Photonics*.

[65] Shaltout A. M., Shalaev V. M., Brongersma M. L. (2019). Spatiotemporal light control with active metasurfaces. *Science*.

[66] Cardin A. E., Silva S. R., Vardeny S. R. (2020). Surface-wave-assisted nonreciprocity in spatio-temporally modulated metasurfaces. *Nat. Commun.*.

[67] Zhang L., Chen X. Q., Shao R. W. (2019). Breaking reciprocity with space-time-coding digital metasurfaces. *Adv. Mater.*.

[68] Taravati S., Khan B. A., Gupta S., Achouri K., Caloz C. (2017). Nonreciprocal nongyrotropic magnetless metasurface. *IEEE Trans. Antenn. Propag.*.

[69] Williamson B. I. A. D., Minkov M., Dutt A., Wang J., Song A. Y., Fan S. (2020). Integrated nonreciprocal photonic devices with dynamic modulation. *Proc. IEEE*.

[70] Gansel J. K., Thiel M., Rill M. S. (2009). Gold helix photonic metamaterial as broadband circular polarizer. *Science*.

[71] Khan M. I., Hu B., Amanat A., Ullah N., Khan M. J. I., Khalid A. R. (2020). Efficient asymmetric transmission for wide incidence angles using bi-layered chiral metasurface. *J. Phys. D Appl. Phys.*.

[72] Zhang L., Zhou P., Chen H. (2016). Ultrabroadband design for linear polarization conversion and asymmetric transmission crossing X- and K- band. *Sci. Rep.*.

[73] Zhao Y., Belkin M. A., Alù A. (2012). Twisted optical metamaterials for planarized ultrathin broadband circular polarizers. *Nat. Commun.*.

[74] Schwanecke A. S., Fedotov V. A., Khardikov V. v., Prosvirnin S. L., Chen Y., Zheludev N. I. (2008). Nanostructured metal film with asymmetric optical transmission. *Nano Lett*..

[75] Plum E., Zhou J., Dong J. (2009). Metamaterial with negative index due to chirality. *Phys. Rev. B: Condens. Matter Mater. Phys.*.

[76] Zhang F., Pu M., Li X. (2017). All-dielectric metasurfaces for simultaneous giant circular asymmetric transmission and wavefront shaping based on asymmetric photonic spin–orbit interactions. *Adv. Funct. Mater.*.

[77] Wang S., Deng Z. L., Wang Y. (2021). Arbitrary polarization conversion dichroism metasurfaces for all-in-one full Poincaré sphere polarizers. *Light. Sci. Appl.*.

[78] Serebryannikov A. E., Magath T., Schuenemann K. (2006). Bragg transmittance of s -polarized waves through finite-thickness photonic crystals with a periodically corrugated interface. *Phys. Rev. E: Stat. Nonlin. Soft Matter Phys.*.

[79] Serebryannikov A. E. (2009). One-way diffraction effects in photonic crystal gratings made of isotropic materials. *Phys. Rev. B: Condens. Matter Mater. Phys.*.

[80] Serebryannikov A. E., Colak E., Magath T., Ozbay E. (2016). Two types of single-beam deflection and asymmetric transmission in photonic structures without interface corrugations. *J. Opt. Soc. Am. A*.

[81] Xu J., Cheng C., Kang M. (2011). Unidirectional optical transmission in dual-metal gratings in the absence of anisotropic and nonlinear materials. *Opt. Lett*..

[82] Xu P., Lv X., Chen J. (2018). Dichroic optical diode transmission in two dislocated parallel metallic gratings. *Nanoscale Res. Lett.*.

[83] Rodríguez-Ulibarri P., Beruete M., Navarro-Cía M., Serebryannikov A. E. (2013). Wideband unidirectional transmission with tunable sign-switchable refraction and deflection in nonsymmetric structures. *Phys. Rev. B: Condens. Matter Mater. Phys.*.

[84] Qiu J., Xu J. (2019). Terahertz wave asymmetric transmission based on double-layer subwavelength dielectric grating. *Opt. Eng.*.

[85] Xu J., Cheng C., Zheng Z. (2010). Electromagnetic transmission in configurations composed of two one-dimensional perfect electric conductor metal gratings. *Chin. Opt Lett.*.

[86] Li S., rong Huang L., hong Ling Y., bing Liu W., fa Ba C., hui Li H. (2019). High-performance asymmetric optical transmission based on coupled complementary subwavelength gratings. *Sci. Rep.*.

[87] Tang B., Li Z., Liu Z., Callewaert F., Aydin K. (2016). Broadband asymmetric light transmission through tapered metallic gratings at visible frequencies. *Sci. Rep.*.

[88] Ozer A., Kocer H., Kurt H. (2018). Broadband and polarization-independent asymmetric transmission of visible light through a three-dimensional trapezoidal metallic metasurface. *J. Opt. Soc. Am. B*.

[89] Li J., Wu X., Hu Q., Ming Y., Hou Y. (2021). Bidirectional edge asymmetric light transmission in metal/dielectric device based on asymmetric diffraction. *Plasmonics*.

[90] Ghobadi A., Dereshgi S. A., Butun B., Ozbay E. (2017). Ultra-broadband asymmetric light transmission and absorption through the use of metal free multilayer capped dielectric microsphere resonator. *Sci. Rep.*.

[91] Ling Y., Huang L., Hong W. (2017). Asymmetric optical transmission based on unidirectional excitation of surface plasmon polaritons in gradient metasurface. *Opt. Express*.

[92] Wu M., Liao Q. (2020). Highly efficient asymmetric optical transmission based on the gradient metasurface and subwavelength grating. *Jpn. J. Appl. Phys.*.

[93] Xu T., Lezec H. J. (2014). Visible-frequency asymmetric transmission devices incorporating a hyperbolic metamaterial. *Nat. Commun*..

[94] Li X., Ji X., Zhang Y., Fan G. (2020). Photonic crystal with tunable air layers based asymmetric transmission film for space solar power station. *Acta Astronaut.*.

[95] Gundogdu T. F., Gokkavas M., Serebryannikov A. E., Ozbay E. (2021). Evidence of asymmetric beaming in a piecewise-linear propagation channel. *Opt. Lett*..

[96] Kurt H., Yilmaz D., Akosman A. E., Ozbay E. (2012). Asymmetric light propagation in chirped photonic crystal waveguides. *Opt. Express*.

[97] Fei H., Zhang Q., Wu M. (2020). Asymmetric transmission of light waves in a photonic crystal waveguide heterostructure with complete bandgaps. *Appl. Opt*..

[98] Heuck M., Kristensen P. T., Elesin Y., Mørk J. (2013). Improved switching using Fano resonances in photonic crystal structures. *Opt. Lett*..

[99] Rosenberg A., Carter M. W., Casey J. A. (2005). Guided resonances in asymmetrical GaN photonic crystal slabs observed in the visible spectrum. *Opt. Express*.

[100] Gippius N. A., Tikhodeev S. G., Ishihara T. (2005). Optical properties of photonic crystal slabs with an asymmetrical unit cell. *Phys. Rev. B: Condens. Matter Mater. Phys.*.

[101] Fei H. M., Yan S., Wu M. (2020). Photonic crystal with 2-fold rotational symmetry for highly efficient asymmetric transmission. *Opt. Commun*..

[102] Onsager L. (1930). Reciprocal Relations In Irreversible Processes. I. *Phys. Rev.*.

[103] Tepper T., Ross C. A., Dionne G. F. (2004). Microstructure and optical properties of pulsed-laser-deposited iron oxide films. *IEEE Trans. Magn*..

[104] Bai J. G., Lu G. Q., Lin T. (2003). Magneto-optical current sensing for applications in integrated power electronics modules. *Sens. Actuators, A*.

[105] Chen Z., Hang Y., Yang L. (2015). Fabrication and characterization of cerium-doped terbium gallium garnet with high magneto-optical properties. *Opt. Lett*..

[106] Yasuhara R., Snetkov I., Starobor A., Palashov O. (2014). Terbium gallium garnet ceramic-based Faraday isolator with compensation of thermally induced depolarization for high-energy pulsed lasers with kilowatt average power. *Appl. Phys. Lett.*.

[107] Chen Z., Yang L., Wang X., Hang Y. (2016). Wavelength dependence of Verdet constant of Pr doped terbium gallium garnet crystal. *Opt. Mater.*.

[108] Vojna D., Slezák O., Lucianetti A., Mocek T. (2019). Verdet constant of magneto-active materials developed for high-power Faraday devices. *Appl. Sci.*.

[109] Snetkov I. L., Permin D. A., Balabanov S. S., Palashov O. v. (2016). Wavelength dependence of Verdet constant of Tb3+:Y2O3 ceramics. *Appl. Phys. Lett.*.

[110] Yakovlev A. I., Snetkov I. L., Dorofeev V. v., Motorin S. E. (2018). Magneto-optical properties of high-purity zinc-tellurite glasses. *J. Non-Cryst. Solids*.

[111] Furuse H., Yasuhara R. (2017). Magneto-optical characteristics of holmium oxide (Ho_2O_3) ceramics. *Opt. Mater. Express*.

[112] Mironov E. A., Palashov O. v., Karimov D. N. (2019). EuF2-based crystals as media for high-power mid-infrared Faraday isolators. *Scr. Mater*..

[113] Jain P. K., Xiao Y., Walsworth R., Cohen A. E. (2009). Surface plasmon resonance enhanced magneto-optics (SuPREMO): faraday rotation enhancement in gold-coated iron oxide nanocrystals. *Nano Lett*..

[114] Torrado J. F., González-Díaz J. B., González M. U., García-artín A., Armelles G. (2010). Magneto-optical effects in interacting localized and propagating surface plasmon modes. *Opt. Express*.

[115] Banthí J. C., Meneses-Rodriguez D., Garcia F. (2012). High magneto-optical activity and low optical losses in metal-dielectric Au/Co/Au-SiO 2 magnetoplasmonic nanodisks. *Adv. Mater.*.

[116] Floess D., Chin J. Y., Kawatani A. (2015). Tunable and switchable polarization rotation with non-reciprocal plasmonic thin films at designated wavelengths. *Light. Sci. Appl.*.

[117] Temnov V. v., Armelles G., Woggon U. (2010). Active magneto-plasmonics in hybrid metal-ferromagnet structures. *Nat. Photonics*.

[118] Maccaferri N., Zubritskaya I., Razdolski I. (2020). Nanoscale magnetophotonics. *J. Appl. Phys.*.

[119] Belotelov V. I., Doskolovich L. L., Zvezdin A. K. (2007). Extraordinary magneto-optical effects and transmission through metal-dielectric plasmonic systems. *Phys. Rev. Lett.*.

[120] Gusynin V. P., Sharapov S. G. (2005). Unconventional integer quantum hall effect in graphene. *Phys. Rev. Lett.*.

[121] Morimoto T., Hatsugai Y., Aoki H. (2009). Optical hall conductivity in ordinary and graphene quantum hall systems. *Phys. Rev. Lett.*.

[122] Gusynin V. P., Sharapov S. G., Carbotte J. P. (2007). Magneto-optical conductivity in graphene. *J. Phys. Condens. Matter*.

[123] Shimano R., Yumoto G., Yoo J. Y. (2013). Quantum faraday and kerr rotations in graphene. *Nat. Commun.*.

[124] Crassee I., Levallois J., Walter A. L. (2011). Giant Faraday rotation in single- and multilayer graphene. *Nat. Phys*..

[125] Crassee I., Orlita M., Potemski M. (2012). Intrinsic terahertz plasmons and magnetoplasmons in large scale monolayer graphene. *Nano Lett*..

[126] Tymchenko M., Nikitin A. Y., Martín-Moreno L. (2013). Faraday rotation due to excitation of magnetoplasmons in graphene microribbons. *ACS Nano*.

[127] Qin J., Xia S., Jia K. (2018). Enhanced Faraday rotation and magneto-optical figure of merit in gold grating/graphene/silicon hybrid magneto-plasmonic devices. *APL Photonics*.

[128] Da H., Qiu C. W. (2012). Graphene-based photonic crystal to steer giant Faraday rotation. *Appl. Phys. Lett.*.

[129] Cox J. D., García De Abajo F. J. (2019). Nonlinear graphene nanoplasmonics. *Acc. Chem. Res*..

[130] Tamagnone M., Fallahi A., Mosig J. R., Perruisseau-Carrier J. (2014). Fundamental limits and near-optimal design of graphene modulators and non-reciprocal devices. *Nat. Photonics*.

[131] Manzeli S., Ovchinnikov D., Pasquier D., Yazyev O. v., Kis A. (2017). 2D transition metal dichalcogenides. *Nat. Rev. Mater.*.

[132] Guddala S., Kawaguchi Y., Komissarenko F. (2021). All-optical nonreciprocity due to valley polarization pumping in transition metal dichalcogenides. *Nat. Commun.*.

[133] Mak K. F., Lee C., Hone J., Shan J., Heinz T. F. (2010). Atomically thin MoS2: a new direct-gap semiconductor. *Phys. Rev. Lett.*.

[134] Liu X., Galfsky T., Sun Z. (2014). Strong light-matter coupling in two-dimensional atomic crystals. *Nat. Photonics*.

[135] Zeng H., Dai J., Yao W., Xiao D., Cui X. (2012). Valley polarization in MoS 2 monolayers by optical pumping. *Nat. Nanotechnol*..

[136] Cao T., Wang G., Han W. (2012). Valley-selective circular dichroism of monolayer molybdenum disulphide. *Nat. Commun.*.

[137] Jalas D., Petrov A., Eich M. (2013). What is-and what is not-an optical isolator. *Nat. Photonics*.

[138] Sounas D. L., Alù A. (2018). Fundamental bounds on the operation of Fano nonlinear isolators. *Phys. Rev. B*.

[139] del Bino L., Silver J. M., Woodley M. T. M., Stebbings S. L., Zhao X., Del’Haye P. (2018). Microresonator isolators and circulators based on the intrinsic nonreciprocity of the Kerr effect. *Optica*.

[140] Xu Y., Miroshnichenko A. E. (2014). Reconfigurable nonreciprocity with a nonlinear Fano diode. *Phys. Rev. B: Condens. Matter Mater. Phys.*.

[141] Mingaleev S. F., Kivshar Y. S. (2002). Photonic-crystal waveguides. ..

[142] Suchkov S. v., Sukhorukov A. A., Huang J., Dmitriev S. v., Lee C., Kivshar Y. S. (2016). Nonlinear switching and solitons in PT-symmetric photonic systems. *Laser. Photon. Rev.*.

[143] Rüter C. E., Makris K. G., El-Ganainy R., Christodoulides D. N., Segev M., Kip D. (2010). Observation of parity-time symmetry in optics. *Nat. Phys*..

[144] Chang L., Jiang X., Hua S. (2014). Parity-time symmetry and variable optical isolation in active-passive- coupled microresonators. *Nat. Photonics*.

[145] Peng B., Ozdemir S. K., Lei F. (2014). Parity-time-symmetric whispering-gallery microcavities. *Nat. Phys*..

[146] Butakov N. A., Valmianski I., Lewi T. (2018). Switchable plasmonic-dielectric resonators with metal-insulator transitions. *ACS Photonics*.

[147] Eyert V. (2002). The metal-insulator transitions of VO2 : A band theoretical approach. *Ann. Phys.*.

[148] Yang Z., Ko C., Ramanathan S. (2011). Oxide electronics utilizing ultrafast metal-insulator transitions. *Annu. Rev. Mater. Res*..

[149] Hafezi M., Rabl P. (2012). Optomechanically induced non-reciprocity in microring resonators. *Opt. Express*.

[150] Hossein-Zadeh M., Vahala K. J. (2007). Observation of optical spring effect in a microtoroidal optomechanical resonator. *Opt. Lett.*.

[151] Cullen A. (1958). A travelling-wave parametric amplifier. *Nature*.

[152] Taravati S., Chamanara N., Caloz C. (2017). Nonreciprocal electromagnetic scattering from a periodically space-time modulated slab and application to a quasisonic isolator. *Phys. Rev. B*.

[153] Taravati S., Eleftheriades G. v. (2020). Full-duplex nonreciprocal beam steering by time-modulated phase-gradient metasurfaces. *Phys. Rev. Appl.*.

[154] Taravati S., Eleftheriades G. v. (2021). Programmable nonreciprocal meta-prism. *Sci. Rep.*.

[155] Wang X., Díaz-Rubio A., Li H., Tretyakov S. A., Alù A. (2020). Theory and design of multifunctional space-time metasurfaces. *Phys. Rev. Appl.*.

[156] Caloz C., Deck-Leger Z. L. (2020). Spacetime metamaterials-Part II: theory and applications. *IEEE Trans. Antenn. Propag.*.

[157] Kamal A. K. (1960). A parametric device as a nonreciprocal element. *Proc. IRE*.

[158] Qin S., Xu Q., Wang Y. E. (2014). Nonreciprocal components with distributed modulated capacitors. *IEEE Trans. Microw. Theor. Tech.*.

[159] Estep N. A., Sounas D. L., Soric J., Alù A. (2014). Magnetic-free non-reciprocity and isolation based on parametrically modulated coupled-resonator loops. *Nat. Phys*..

[160] Reiskarimian N., Krishnaswamy H. (2016). Magnetic-free non-reciprocity based on staggered commutation. *Nat. Commun.*.

[161] Dinc T., Tymchenko M., Nagulu A., Sounas D., Alu A., Krishnaswamy H. (2017). Synchronized conductivity modulation to realize broadband lossless magnetic-free non-reciprocity. *Nat. Commun.*.

[162] Nagulu A., Krishnaswamy H. (2019). Non-magnetic CMOS switched-transmission-line circulators with high power handling and antenna balancing: theory and implementation. *IEEE J. Solid State Circ.*.

[163] Nagulu A., Reiskarimian N., Krishnaswamy H. (2020). Non-reciprocal electronics based on temporal modulation. *Nat. Electron.*.

[164] Zang J. W., Correas-Serrano D., Do J. T. S., Liu X., Alvarez-Melcon A., Gomez-Diaz J. S. (2019). Nonreciprocal wavefront engineering with time-modulated gradient metasurfaces. *Phys. Rev. Appl.*.

[165] Mandal A., Cui Y., McRae L., Gholipour B. (2021). Reconfigurable chalcogenide phase change metamaterials: a material, device, and fabrication perspective. *JPhys Photonics*.

[166] Wang Q., Rogers E. T. F., Gholipour B. (2016). Optically reconfigurable metasurfaces and photonic devices based on phase change materials. *Nat. Photonics*.

[167] Karvounis A., Gholipour B., MacDonald K. F., Zheludev N. I. (2016). All-dielectric phase-change reconfigurable metasurface. *Appl. Phys. Lett.*.

[168] Gholipour B., Zhang J., MacDonald K. F., Hewak D. W., Zheludev N. I. (2013). An all-optical, non-volatile, bidirectional, phase-change meta-switch. *Adv. Mater.*.

[169] Gholipour B., Piccinotti D., Karvounis A., Macdonald K. F., Zheludev N. I. (2019). Reconfigurable ultraviolet and high-energy visible dielectric metamaterials. *Nano Lett*..

[170] Bernevig B. Andrei (2013). ..

[171] Ozawa T., Price H. M., Amo A. (2019). Topological photonics. *Rev. Mod. Phys.*.

[172] Khanikaev A. B., Shvets G. (2017). Two-dimensional topological photonics. *Nat. Photonics*.

[173] Haldane F. D. M., Raghu S. (2008). Possible realization of directional optical waveguides in photonic crystals with broken time-reversal symmetry. *Phys. Rev. Lett.*.

[174] Ao X., Lin Z., Chan C. T. (2009). One-way edge mode in a magneto-optical honeycomb photonic crystal. *Phys. Rev. B: Condens. Matter Mater. Phys.*.

[175] Wang Z., Chong Y., Joannopoulos J. D., Soljačić M. (2009). Observation of unidirectional backscattering-immune topological electromagnetic states. *Nature*.

[176] Fang K., Yu Z., Fan S. (2012). Realizing effective magnetic field for photons by controlling the phase of dynamic modulation. *Nat. Photonics*.

[177] Rechtsman M. C., Zeuner J. M., Plotnik Y. (2013). Photonic Floquet topological insulators. *Nature*.

[178] Kane C. L., Mele E. J. (2005). Quantum Spin hall effect in graphene. *Phys. Rev. Lett.*.

[179] Kane C. L., Mele E. J. (2005). Z2 topological order and the quantum spin hall effect. *Phys. Rev. Lett.*.

[180] Hafezi M., Demler E. A., Lukin M. D., Taylor J. M. (2011). Robust optical delay lines with topological protection. *Nat. Phys*..

[181] Liang G. Q., Chong Y. D. (2013). Optical resonator analog of a two-dimensional topological insulator. *Phys. Rev. Lett.*.

[182] Khanikaev A. B., Hossein Mousavi S., Tse W. K., Kargarian M., MacDonald A. H., Shvets G. (2013). Photonic topological insulators. *Nat. Mater*..

[183] Chen W. J., Jiang S. J., Chen X. D. (2014). Experimental realization of photonic topological insulator in a uniaxial metacrystal waveguide. *Nat. Commun.*.

[184] Anderson P. D., Subramania G. (2017). Unidirectional edge states in topological honeycomb-lattice membrane photonic crystals. *Opt. Express*.

[185] Wu L. H., Hu X. (2015). Scheme for achieving a topological photonic crystal by using dielectric material. *Phys. Rev. Lett.*.

[186] Liu W., Hwang M., Ji Z., Wang Y., Modi G., Agarwal R. (2020). Z2 photonic topological insulators in the visible wavelength range for robust nanoscale photonics. *Nano Lett*..

[187] Harari G., Bandres M. A., Lumer Y. (2018). Topological insulator laser: theory. *Science*.

[188] Lu L., Joannopoulos J. D., Soljačić M. (2014). Topological photonics. *Nat. Photonics*.

[189] Wu J., Ng B., Liang H. (2014). Chiral metafoils for terahertz broadband high-contrast flexible circular polarizers. *Phys. Rev. Appl.*.

[190] Fedotov V. A., Schwanecke A. S., Zheludev N. I., Khardikov V. v., Prosvirnin S. L. (2007). Asymmetric transmission of light and enantiomerically sensitive plasmon resonance in planar chiral nanostructures. *Nano Lett*..

[191] Ma D., Li Z., Zhang Y. (2019). Giant spin-selective asymmetric transmission in multipolar-modulated metasurfaces. *Opt. Lett*..

[192] Stephen L., Yogesh N., Subramanian V. (2018). Broadband asymmetric transmission of linearly polarized electromagnetic waves based on chiral metamaterial. *J. Appl. Phys.*.

[193] Menzel C., Helgert C., Rockstuhl C. (2010). Asymmetric transmission of linearly polarized light at optical metamaterials. *Phys. Rev. Lett.*.

[194] Frese D., Wei Q., Wang Y., Huang L., Zentgraf T. (2019). Nonreciprocal asymmetric polarization encryption by layered plasmonic metasurfaces. *Nano Lett*..

[195] Zhang C., Pfeiffer C., Jang T. (2016). Breaking Malus’ law: highly efficient, broadband, and angular robust asymmetric light transmitting metasurface. *Laser. Photon. Rev.*.

[196] Shadrivov I. v., Fedotov V. A., Powell D. A., Kivshar Y. S., Zheludev N. I. (2011). Electromagnetic wave analogue of an electronic diode. *New J. Phys.*.

[197] Kenanakis G., Xomalis A., Selimis A. (2015). Three-dimensional infrared metamaterial with asymmetric transmission. *ACS Photonics*.

[198] Whiting E. B., Goldflam M. D., Kang L. (2022). Broadband asymmetric transmission of linearly polarized mid-infrared light based on quasi-3D metamaterials. *Adv. Funct. Mater.*.

[199] Balthasar Mueller J. P., Rubin N. A., Devlin R. C., Groever B., Capasso F. (2017). Metasurface polarization optics: independent phase control of arbitrary orthogonal states of polarization. *Phys. Rev. Lett.*.

[200] Yao Y., Liu H., Wang Y. (2016). Nanoimprint-defined, large-area meta-surfaces for unidirectional optical transmission with superior extinction in the visible-to-infrared range. *Opt. Express*.

[201] Serebryannikov A. E., Ozbay E. (2009). Unidirectional transmission in non-symmetric gratings containing metallic layers. *Opt. Exp.*.

[202] Ye W.-M., Yuan X.-D., Guo C.-C., Zen C. (2010). Unidirectional transmission in non-symmetric gratings made of isotropic material. *Opt. Exp.*.

[203] Serebryannikov A. E., Ozbay E., Nojima S. (2014). Asymmetric transmission of terahertz waves using polar dielectrics. *Opt. Express*.

[204] Foteinopoulou S. (2022). Breaking transmission symmetry without breaking reciprocity in linear all-dielectric polarization-preserving metagratings. *Phys. Rev. Appl.*.

[205] Lockyear M. J., Hibbins A. P., White K. R., Sambles J. R. (2006). One-way diffraction grating. *Phys. Rev. E: Stat. Nonlin. Soft Matter Phys.*.

